# A Conceptual Model Relationship between Industry 4.0—Food-Agriculture Nexus and Agroecosystem: A Literature Review and Knowledge Gaps

**DOI:** 10.3390/foods13010150

**Published:** 2024-01-01

**Authors:** Chee Kong Yap, Khalid Awadh Al-Mutairi

**Affiliations:** 1 Department of Biology, Faculty of Science, Universiti Putra Malaysia, Serdang 43400 UPM, Selangor, Malaysia; 2 Department of Biology, Faculty of Science, University of Tabuk, Tabuk P.O. Box 741, Saudi Arabia; kmutairi@ut.edu.sa

**Keywords:** Industry 4.0, agriculture, food industry, social, economy, environment

## Abstract

With the expected colonization of human daily life by artificial intelligence, including in industry productivity, the deployment of Industry 4.0 (I4) in the food agriculture industry (FAI) is expected to revolutionize and galvanize food production to increase the efficiency of the industry’s production and to match, in tandem, a country’s gross domestic productivity. Based on a literature review, there have been almost no direct relationships between the I4—Food-Agriculture (I4FA) Nexus and the agroecosystem. This study aimed to evaluate the state-of-the-art relationships between the I4FA Nexus and the agroecosystem and to discuss the challenges in the sustainable FAI that can be assisted by the I4 technologies. This objective was fulfilled by (a) reviewing all the relevant publications and (b) drawing a conceptual relationship between the I4FA Nexus and the agroecosystem, in which the I4FA Nexus is categorized into socio-economic and environmental (SEE) perspectives. Four points are highlighted in the present review. First, I4 technology is projected to grow in the agricultural and food sectors today and in the future. Second, food agriculture output may benefit from I4 by considering the SEE benefits. Third, implementing I4 is a challenging journey for the sustainable FAI, especially for the small to medium enterprises (SMEs). Fourth, environmental, social, and governance (ESG) principles can help to manage I4’s implementation in agriculture and food. The advantages of I4 deployment include (a) social benefits like increased occupational safety, workers’ health, and food quality, security, and safety; (b) economic benefits, like using sensors to reduce agricultural food production costs, and the food supply chain; and (c) environmental benefits like reducing chemical leaching and fertilizer use. However, more studies are needed to address social adaptability, trust, privacy, and economic income uncertainty, especially in SMEs or in businesses or nations with lower resources; this will require time for adaptation to make the transition away from human ecology. For agriculture to be ESG-sustainable, the deployment of I4FA could be an answer with the support of an open-minded dialogue platform with ESG-minded leaders to complement sustainable agroecosystems on a global scale.

## 1. Introduction

The topics of Agriculture-Industry 4.0 (I4) [1,2,3,4,5,6,7,8,9,10,11,12,13,14,15,16,17,18,19,20,21] and Foods-I4 [22,23,24,25,26,27,28,29,30,31,32,33,34,35,36,37,38,39,40,41,42,43,44,45,46,47,48,49,50,51,52,53,54,55,56,57,58,59,60,61,62,63,64,65,66,67,68,69,70,71,72,73,74,75,76,77,78,79,80,81,82,83,84,85,86,87,88,89] can be found in the literature. Additionally, precision agriculture (PA), which incorporates elements of I4, has also been widely reported [90,91,92,93,94,95,96,97,98,99,100,101,102,103,104,105,106,107,108,109,110,111,112,113,114,115,116,117,118,119,120,121,122,123,124,125,126,127,128,129,130,131,132,133,134,135,136,137,138,139,140,141,142,143,144,145,146,147,148,149,150,151,152,153,154,155,156,157,158,159,160,161,162,163,164]. However, there has been a lack of discussion on the direct relationships between the I4—Food-Agriculture (I4FA) Nexus and the agroecosystem.

Before this review paper discusses the above topic of concern in Section 5, the basic understanding of the agroecosystem [165,166,167,168], I4 for PA [90,91,92,93,94,95,96,97,98,99,100,101,102,103,104,105,106,107,108,109,110,111,112,113,114,115,116,117,118,119,120,121,122,123,124,125,126,127,128,129,130,131,132,133,134,135,136,137,138,139,140,141,142,143,144,145,146,147,148,149,150,151,152,153,154,155,156,157,158,159,160,161,162,163,164,169,170,171,172], and I4 for current and future sustainable food agriculture [20,173,174,175] is introduced in the following opening sections.

### 1.1. The Agroecosystem

**Figure 1:**
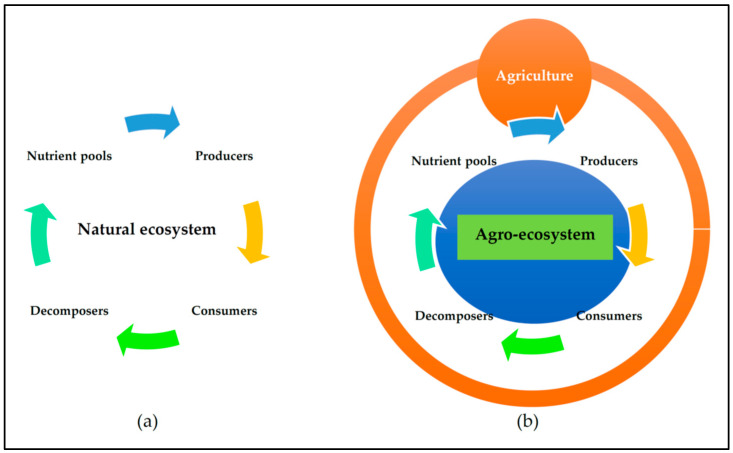
Four basic components between (**a**) a natural ecosystem (where there are renewable natural resources from the ecological ecosystem) and (**b**) an agroecosystem (where all nutrients and planting conditions are regulated and controlled by man) [165,166].

An ecosystem’s existence is and should be supported by nutrient cycling, both spatially and temporally. In an ecosystem, heterotrophs, autotrophs, and decomposers (microbes) recycle nutrients from nutrient pools (Figure 1). Thus, their interactions make up a basic ecosystem’s functioning. Overall, the nutrient–producer–consumer–decomposer nexus can be considered the mother of sustainability. The agroecosystem is no exception to the addition of human activities.

The factors of human control and net productivity are higher in agroecosystems when compared to those in the natural ecosystem. However, species, genetic diversity, and stability are higher in the natural ecosystem when compared to those in the agroecosystem. Furthermore, the trophic interactions and habitat heterogeneity are simple (or linear) in agroecosystems, whereas in the natural ecosystem they are complicated (or complex). The nutrient cycles are a closed system in the natural ecosystem, but the agroecosystem is an open one [165,166]. Agroecosystems are coexisting human–natural production systems that supply the rising human population’s need for food, fuel, and fiber [165,166,167,168]. Agroecosystems are also frequently linked to higher nutrient input, most of which escapes from the farming area and thus causes eutrophication in nearby ecosystems that are not directly involved in agriculture. The common question is, “How can we sustain the agroecosystem to cater to increasing food demand and security?” The significant challenges now are the changes brought about by the natural and human-induced processes that impact how they can operate sustainably in a nutrient-cycling model between a natural ecosystem and a man-impacted agroecosystem (Figure 1).

### 1.2. Industry 4.0 for Precision Agriculture

Industrial revolutions are technological developments that impact society, development, and the environment. The steam engine and broad energy availability started the first industrial revolution; the assembly line and mass manufacturing started the second; and robots to perform work started the third [8]. The fourth industrial revolution is being discussed (Figure S1). The production systems should speak informally and make decisions based on system facts. I4 involves digitization, food supply chain (FSC) management analytics tools for monitoring, tracking, and analysis, and operational competence and efficiency. I4 accelerates assembly digitalization by employing sensors and other electronics across all assembly segments and products [15].

Sustainable business practices in energy-efficient building and smart manufacturing with low-carbon emission industrialization are supported by I4 technology [169]. Since 2020, an increasing number of articles have related I4 to sustainability [169,170,171,172]. The rising research linking I4 and sustainability shows that smart factories are built on sustainability [172]. In the future, I4 technologies will be widely used in socio-economic and environmental (SEE) sectors. This entails developing and improving innovative digital tools and instruments for the massive data collecting driven by unforeseen industry developments [1,2,3]. Multiple I4 sustainability functions have complicated the preceding linkages, according to Ghobakhloo [170]. I4’s immediate outcomes prepare the way for its socio-environmental sustainability functions, such as increased social welfare, sustainable energy, and harmful emissions reduction. Ghobakhloo [170] also believed the digital revolution would promote sustainability. Thus, they worked together to guarantee that I4.0 effectively, reasonably, and equally fulfilled global sustainability plans.

Mobile technology significantly influences sustainability in all industries, whereas nanotechnology significantly impacts cars and electronics [171]. Technical, social, and structural development and networked and cooperative digitalization are expected in I4 [10]. High agricultural production is needed to meet the growing food demand. Cyber–physical systems (CPSs), the Internet of Services (IoS), the Internet of Things (IoT), cloud computing (CC), and big data are I4 technologies that might digitize agricultural FSC [17]. I4 is the most notable technological innovation that might assist businesses and entrepreneurs in meeting these challenges [8].

### 1.3. Industry 4.0 for Current and Future Sustainable Food Agriculture

The literature on sustainable agriculture can be found [90,91,92,93,94,95,96,97,98,99,100,101,102,103,104,105,106,107,108,109,110,111,112,113,114,115,116,117,118,119,120,121,122,123,124,125,126,127,128,129,130,131,132,133,134,135,136,137,138,139,140,141,142,143,144,145,146,147,148,149,150,151,152,153,154,155,156,157,158,159,160,161,162,163,164]. However, not all publications mention the use of I4 directly. However, the elements of I4 are proposed indirectly and are already implemented in PA’s sustainability [90,91,92,93,94,95,96,97,98,99,100,101,102,103,104,105,106,107,108,109,110,111,112,113,114,115,116,117,118,119,120,121,122,123,124,125,126,127,128,129,130,131,132,133,134,135,136,137,138,139,140,141,142,143,144,145,146,147,148,149,150,151,152,153,154,155,156,157,158,159,160,161,162,163,164].

Farmers should expect more profits from PA technology. PA should improve society’s sustainability [93]. PA is growing more popular worldwide as a dynamic manufacturing method. In assessing its environmental and economic sustainability, this approach’s ability to reduce pesticide use by controlling land parcel-level pesticide application and boosting profitability and incomes was considered. PA has been linked to social collective action, but little is known regarding the actor and education roles [96].

The agriculture sustainability issues include nitrogen management. PA approaches instead of regular tillage may boost nitrogen cycle efficiency, benefiting the environment, crops, and soils [109]. Nanomaterials in agriculture are used in crop production, soil and water management, diagnostic measures, controlled chemical usage, and plant protection due to their properties, tiny size, and surface-to-volume ratio [142]. PA’s usage of nanotechnology advanced with nano-based insecticides, herbicides, fertilizers, and early disease diagnoses [142]. The major method for ensuring the sustenance and economic growth of a nation is agriculture. PA’s rapid advancement has helped agriculture and related industries to adapt to big data and machine intelligence. Machine learning offers useful analytical and computational approaches for integrating datasets from several sources [149].

The two fundamental agricultural concerns consist of the growing of nutritious food while lowering crop production’s negative consequences on the land, water, and climate [115]. Controlling plant infections can help solve these problems since plant diseases reduce crop productivity and profitability, which feeds a large portion of the globe. New methods and technology are needed to sustain agricultural production systems and manage plant diseases [115]. PA advances greener agriculture. Many farmers have the equipment for on-site operation but rarely use it, limiting I4 utilization [119]. Sahoo et al. [152] stated that sustainable agriculture is essential to all life on Earth since the world still needs food. Sustainable agriculture involves holistic livestock, crop, and fisheries management to make farming more self-sustaining over time [152].

Sustainable agriculture using I4 has been reported since 2019 [20,173,174,175]. Trivelli et al. [20] suggested I4 for PA in the agri-food business. I4 technologies may help accomplish the UN SDGs and assist the agricultural FSC [74]. Unmanned vehicles detect insect migration, identify species, estimate damage, and apply pesticides on the spot for precision control in digital agriculture [175]. Smallholder farmers may benefit from the creation of a digital platform that addresses their issues throughout the farming cycle and brings all the relevant parties together at the national level to promote sustainable agriculture and cutting-edge digital technology [173]. Santiteerakul et al. [174] stated that a plant factory using intelligence technology might increase product quality, productivity, crop yield by year, food safety, resource efficiency, and staff quality of life. If the food processing business understands I4, the digital–physical framework will spur global food sector advances. This may inspire all organizations to provide innovative food and develop greater competition around the food agricultural industry (FAI) expansion [74].

According to the literature, I4 technologies will boost agricultural output today and in the future. Hence, they should be linked. Two reasons explain the link.

First, food comes from agriculture. Studies and talks on deploying I4 in agriculture to increase food security for the growing population have been well reported [1,2,3,4,5,6,7,8,9,10,11,12,13,14,15,16,17,18,19,20,21]. That was a smart move. The rising demand for agricultural commodities, notably processed meals, meat, dairy, and seafood, will strain food production and delivery networks. This study examines whether the technologies that underpin these two PA paradigms are similar [20]. Digital technologies have a similar function. Agriculture and allied activities must support all human pursuits for future food security. However, population increase and resource competition continue to threaten agricultural supply networks, threatening sustainable agriculture. To address agricultural sustainability, PA and FSC coordination must improve [9]. These issues are becoming increasingly sophisticated in agricultural supply networks and production systems. I4 for agriculture is likely to be the answer.

Second, I4 and related technologies might make food agriculture firms more competitive in the digital age [4]. Agriculture 4.0 is typified by the growing use of digital technology in food [5]. Agriculture and livestock are vital to social and economic stability. FSC management benefits from increased visibility, provenance, digitalization, disintermediation, and smart contracts [62]. Prasad et al. [3] reported that the IoT links many items, technologies, and devices in a network to speed up processes, eliminate information loss, and enable device–cloud/device–device communication. The fundamental question is how IoT will assist food and agriculture. I4 smart agriculture uses IoT in urban and rural areas [4].

As there is a lack of study on the direct relationships between the I4FA Nexus and the agroecosystem, the objectives of this study are (a) to evaluate the state-of-the-art relationships between the I4FA Nexus and the agroecosystem and (b) to discuss the challenges and knowledge gaps in the sustainable FAI that the I4 technologies can assist. The purpose is fulfilled by (a) reviewing all the relevant publications from the Scopus database and (b) drawing a conceptual relationship between the I4FA Nexus and the agroecosystem, in which the I4FA Nexus is categorized into economic, societal, and environmental perspectives.

## 2. Methodology

### Literature Collection

**Figure 2:**
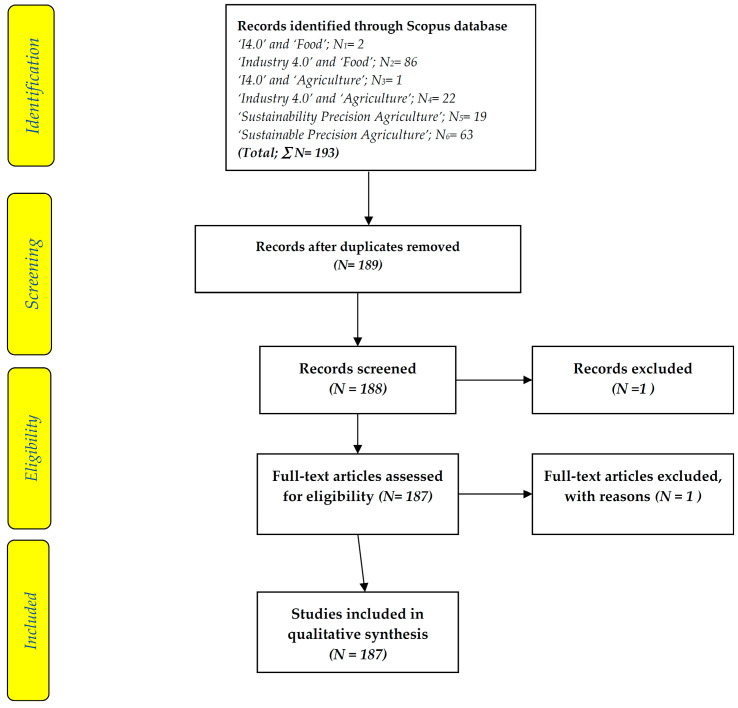
Flowchart of Preferred Reporting Items for Systematic Reviews and Meta-Analyses (PRISMA) used in the present study, adapted from Moher et al. [176].

Instead of a wide standard literature review, a systematic literature review (SLR) is more suitable. Thus, in the current review study, the SLR technique of Preferred Reporting Items for Systematic Reviews and Meta-Analyses (PRISMA) by Moher et al. [176] was employed to add to the body of information already available in “Industry 4.0” and “Food”. The evidence-based reporting standard of PRISMA is helpful for critical evaluation. Overall, Figure 2 depicts the systematic process stages that were modified for this review paper. As Elsevier‘s Scopus is the world’s largest abstract and citation database of peer-reviewed scientific literature journals, books, and conference proceedings and covers research topics across all scientific, technical, and medical disciplines [177], it was chosen for the literature analysis in the present study.

This study assessed the scholarly distributions on “I4.0” and “Food” found in the Scopus bibliographic database, which was chosen for its size and the variety of its distributions. On 10 December 2023, by using the keywords ‘I4.0’ or ‘Industry 4.0’ and ‘Food’, which must be found in the title of the papers under the Scopus database, a total of 88 papers arrived. After removing 4 duplicated papers and 1 irrelevant paper, a total of 84 papers from the Scopus database were found. With the keywords ‘Industry 4.0’, or ‘I4.0’ and ‘Agriculture’, a total of 23 papers were found (Figure 2).

In addition, the topics on ‘sustainability (or sustainable) precision agriculture’, which had to appear in the article title, were found in 82 papers, of which 19 papers had the keywords ‘sustainability precision agriculture’, and 63 papers had ‘sustainable precision agriculture’ (one was discarded due to its being a ‘correction’ article). Therefore, a total of 187 papers are included in the present review study (Figure 2).

Bibliometric analyses are an established method to evaluate research literature, particularly in the scientific fields that benefit from computational data treatment and that have witnessed increased scholarly output [178]. VOSviewer (version 1.6.20) is software that generates a clear graphical representation of bibliometric maps, especially for extensive datasets [179]. To highlight the trends of the studies conducted on the topic of ‘Industry 4.0’ and ‘Food’ from 2016 to 2024 (on 111 papers from the Scopus database), we performed a bibliometric analysis using the VOSviewer software (VOS stands for visualization of similarities—see www.vosviewer.com; accessed on 5 December 2023). Separately, other visualizations were performed based on ‘sustainability (or sustainable) precision agriculture’ from 1995 to 2023 (on 82 papers from the Scopus database). Scopus comprises many significant research papers and offers integrated analysis tools for creating informative visual representations [177]. VOSviewer was employed to analyze each keyword, calculating links, total link strengths, and co-occurrences with other keywords.

## 3. Results

The studies and discussions on the use of I4 in the food sectors in particular have been reported in the literature [22,23,24,25,26,27,28,29,30,31,32,33,34,35,36,37,38,39,40,41,42,43,44,45,46,47,48,49,50,51,52,53,54,55,56,57,58,59,60,61,62,63,64,65,66,67,68,69,70,71,72,73,74,75,76,77,78,79,80,81,82,83,84,85,86,87,88,89] (with a total of 67 papers) (Figure 2) and represent significantly more in terms of the number of publications than ‘I4’ plus ‘agriculture’ per se [1,2,3,4,5,6,7,8,9,10,11,12,13,14,15,16,17,18,19,20,21] (with a total of 21 papers). This is because food items are part and parcel of the human needs and life requirements that are necessary for the continued survival of humankind. At the same time, agriculture is the center of activities where human food is provided. There are 14 countries/regions (Table S1) selected based on the relevancy of adopting I4 into the FAI, based on a literature search on the keywords ‘I4’ or ‘Industry 4.0’ and ‘Food’ found in different regions or countries.

After carefully examining each paper, the reviewed articles can be specifically categorized based on the focus of the studies/review. The order of the decreasing number of the categories is socio-economy > SEE > social > sustainability > economy > socio-environment > economic environment > environment [1]. This indicates that the reviewed papers with the I4FA Nexus are mainly concerned with SEE and sustainability. The following discussion is therefore weighted on the social, economic, and environmental categories under the I4FA Nexus.

**Figure 3:**
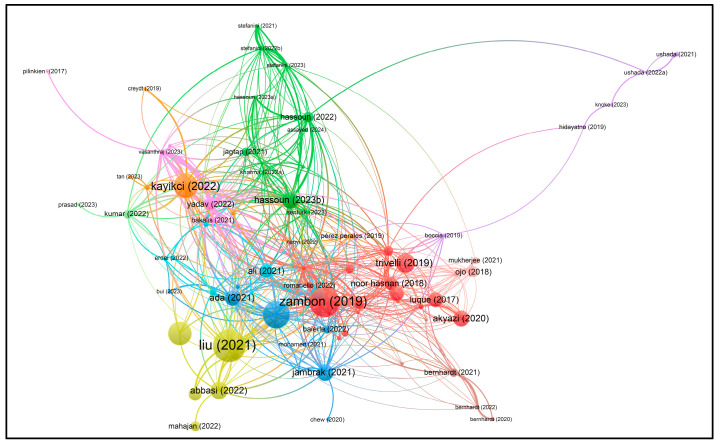
A bibliometric analysis of research that created a visualization of the paper network confirmed the main themes of research based on 84 papers (documents) (out of 111 papers) with 11 clusters. The literature is based on the Scopus database; the keywords ‘I4.0’ or ‘Industry 4.0’ and ‘Food’, which had to appear in the article title, were found in 111 papers. The papers ranged from 2016 to 2024 and 2019 to 2023.

**Figure 4:**
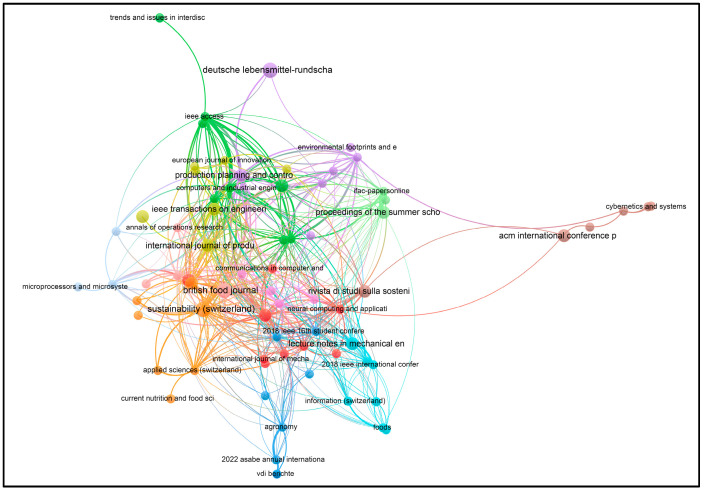
A bibliometric analysis of research that created a visualization of the paper network confirming the main themes of research, based on 72 journals (sources) with 12 clusters. The literature is based on the Scopus database; the keywords ‘I4.0’ or ‘Industry 4.0’ and ‘Food’, which had to appear in the article title, were found in 111 papers. The papers ranged from 2016 to 2024 and 2019 to 2023.

**Figure 5:**
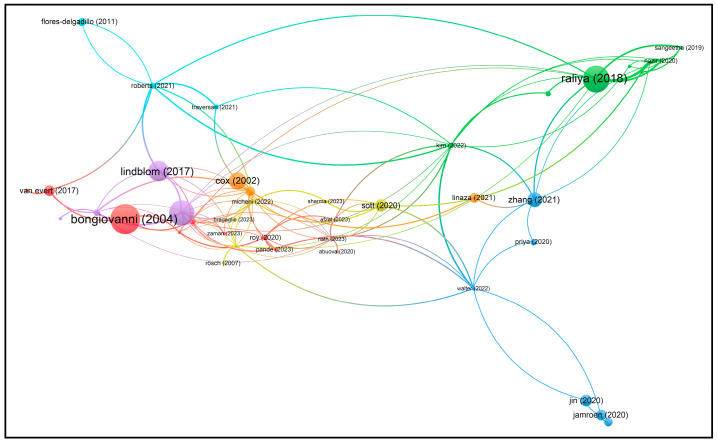
A bibliometric analysis of research created a visualization of the paper network confirming the main research themes based on 43 papers (documents) with 8 clusters. The literature is based on the Scopus database on the topics of ‘sustainability (or sustainable) precision agriculture’, which had to appear in the article title; 82 papers were found. The papers ranged from 1995 to 2023 and 2002 to 2023.

**Figure 6:**
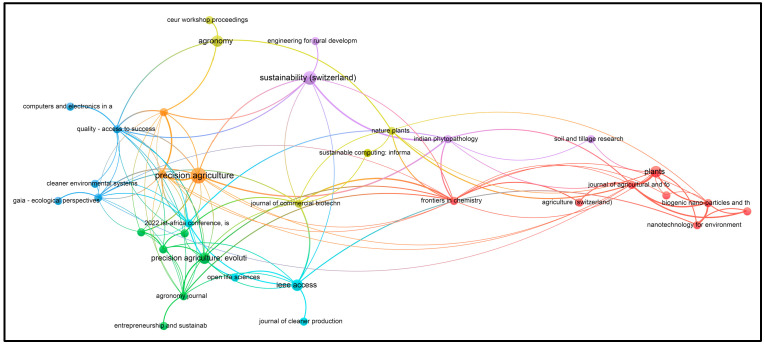
A bibliometric analysis of research created a visualization of the paper network confirming the main research themes based on 35 sources (journals) with 7 clusters. The literature is based on the Scopus database on the topics of ‘sustainability (or sustainable) precision agriculture’, which had to appear in the article title; 82 papers were found. The papers ranged from 1995 to 2023 and 2002 to 2023.

Using VOSviewer software, visualizations of the paper network-based data confirmed the main themes of research based on the documents and sources using clustering patterns, which are presented in Figure 3, Figure 4, Figure 5 and Figure 6.

Based on the keywords ‘I4’ and ‘Food’, the authors have mainly published their papers since 2018, according to the visualization (Figure 3). This indicates increasing numbers of papers, sometimes with similar authors or co-authors, specializing in similar topics to satisfy the current and future knowledge needs regarding I4 and food.

From Figure 4, the visualization shows that at least 72 different journals have been published on the topics of ‘I4’ and ‘Food’ since 2016. The journals include *Sustainability* (Basel), *British Food Journal*, *Applied Sciences*, *Information*, *ACM International Conference Proceeding Series*, *Advances in Intelligent Systems and Computing*, *Deutsche Lebensmittel-Rundschau*, *E3S Web of Conferences*, *Engineering Proceedings*, and others, as shown in Table S2.

Based on the keywords ‘sustainability (or sustainable) precision agriculture’, the authors have mainly published their papers since 2002, according to the visualization (Figure 5). This indicates increasing numbers of papers, sometimes with similar authors or co-authors, specializing in similar topics to satisfy the current and future knowledge needs regarding sustainable agriculture.

In Figure 6, the visualization shows that at least 35 different journals have been published on the topics of ‘sustainability (or sustainable) precision agriculture’ since 2002. The journals include *Agriculture* (Switzerland), *Agronomy Journal*, *American Journal of Alternative Agriculture*, *Biochemical Systematics and Ecology*, *Biomaterials Advances*, *Biosystems Engineering*, and others, as shown in Table S3.

## 4. Discussion

The following discussions will focus on the four major observations based on the literature reviewed in the present study. They are: Section 4.1. The adoption of I4 in the agriculture and food sectors has been constantly growing since 2011 and is expected to increase in the future; Section 4.2. Good prospects for the I4 implementation into food agricultural production; Section 4.3. The challenges of the sustainable agricultural food industry in adopting Industry 4.0; and Section 4.4. The knowledge gaps for future studies.

### 4.1. The Adoption of I4 in the Agriculture and Food Sectors Has Been Constantly Growing since 2011 and Is Expected to Increase in the Future

This is well supported by the literature review from three critical points, namely: (a) the expected higher number of publications on the topics of I4 food agricultural production in the future; (b) the social perspective on the growth of the human population; and (c) the number of countries that have started using I4 in their food agricultural industries.

#### 4.1.1. Expectedly Higher Number of Publications on the Topics of I4 Food Agricultural Production in the Future

**Figure 7:**
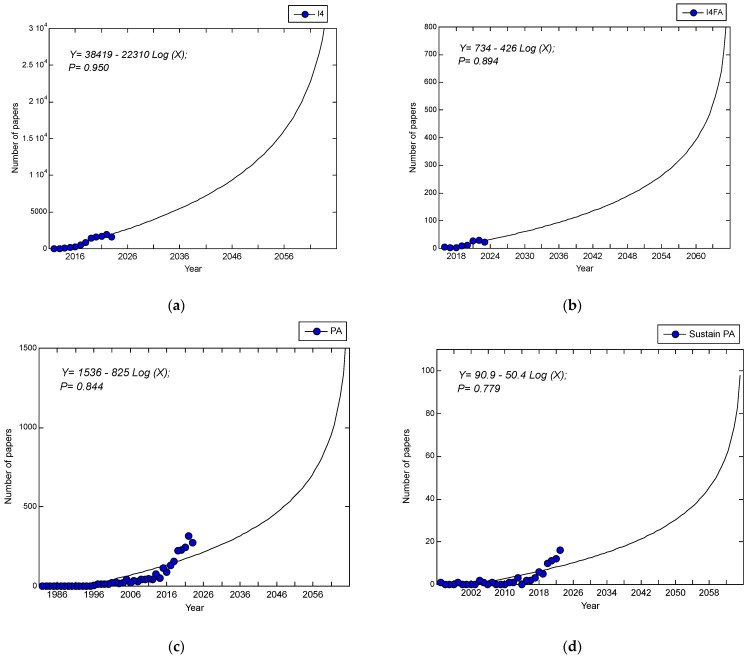
Numbers of papers with keywords of (**a**) ‘Industry 4.0’ (I4; from 2012 to 2023); (**b**) ‘Industry 4.0 Food Agriculture’ (I4FA; from 2016 to 2023); (**c**) ‘Precision Agriculture’ (PA; from 1982 to 2023); (**d**) ‘Sustainable (or sustainability) Precision Agriculture (Sustain PA; from 1995 to 2023), based on Scopus database. In addition, the number of papers is extrapolated to 2065 using logarithmic equations for the four graphs. Note: 1 10^4^ indicates 1 × 10^4^; similarly applying to others.

It is expected that a higher number of publications started earlier on the topic of I4 (since 2012) (Figure 7a) than on the topic of I4FA (since 2016) (Figure 7b). It is expected that the number of publications on I4 will increase to over 30,000 papers by 2065 (Figure 7a), while that on I4FA will reach over 800 papers by 2065 (Figure 7b). This is logically acceptable, considering that the I4 topic covers all study disciplines, ranging from sea to land to outer space. It is interesting to see that a higher number of publications started earlier on the topic of PA (since 1982) (Figure 7c) than on the topic of sustainable (or sustainability) PA (since 1995) (Figure 7d). It is expected that the number of publications on PA will increase to over 1500 papers by 2065 (Figure 7c), while that on sustainable (or sustainability) PA will reach over 100 papers by 2065 (Figure 7d). Notably, when the keyword ‘Sustainable’ (or ‘Sustainability’) is included in the topic of a scientific paper, the specialization of a niche discipline of a reach study is triggered, with ample potential for research topics and opportunities. Moreover, the UNSDG timeframe is only until 2030, but the sustainability effort in SEE is continuous. The UNSGDs’ 2030 deadline needs an extension to an unlimited time frame when climate change is taken into consideration.

**Figure 8:**
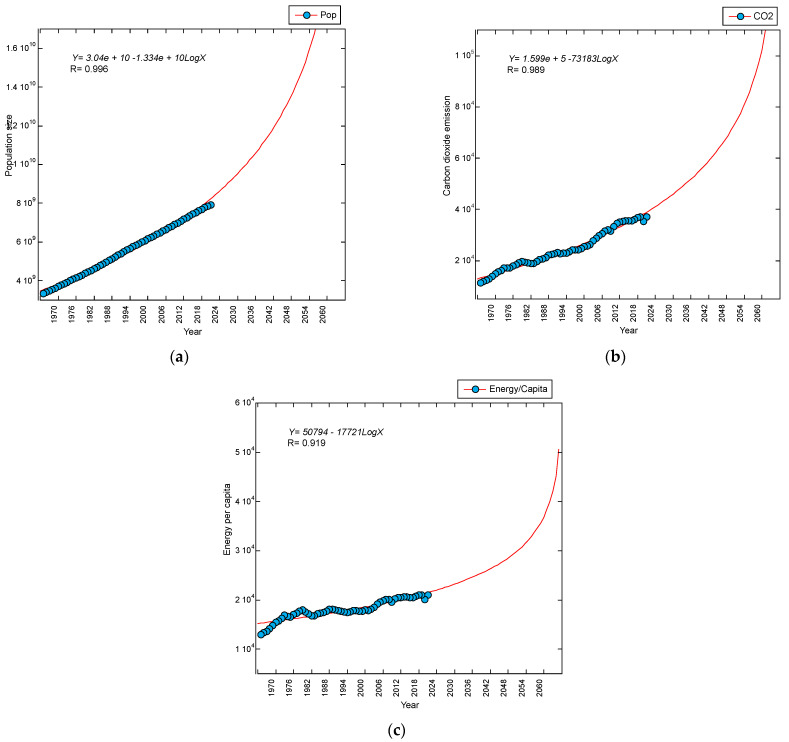
Increasing trends from 1965 to October 2023 for (**a**) ‘Population size’ (Pop), (**b**) ‘carbon dioxide emission’ (CO_2_), and (**c**) ‘Energy per capita’ from 1965 to October 2023, based on data cited from the OurWorldInData.org. In addition, their increasing trends are extrapolated to 2065 using logarithmic equations for the three graphs. Note: Annual total production-based carbon dioxide (CO_2_) emissions, excluding land-use change, measured in million tonnes. Primary energy consumption per capita (energy per capita), measured in kilowatt-hours per person per year. 1 10^4^ indicates 1 × 10^4^; similarly applying to others.

These positive increasing trends are in line with those of (a) ‘Population size’ (Pop), (b) ‘carbon dioxide emission’ (CO_2_), and (c) ‘Energy per capita’ from the same periods, based on data cited from the OurWorldInData.org, as shown Figure 8. This expected higher number of publications on the topics of I4 food agricultural production in the future contributes to the massive paradigm shift of I4 implementation in the future of food agricultural production, which is discussed in terms of the social aspect in Section 4.1.2 below.

#### 4.1.2. Social Perspective on the Increment of the Human Population

The positive increasing trend could be explained from a social perspective. There have been increasing numbers of countries employing I4 in their food agricultural activities to cost-effectively supply the increasing demand for food among their increasing population sizes (Figure 8a). The following papers [22,23,24,25,26,27,28,29,30,31,32,33,34,35,36,37,38,39,40,41,42,43,44,45,46,47,48,49,50,51,52,53,54,55,56,57,58,59,60,61,62,63,64,65,66,67,68,69,70,71,72,73,74,75,76,77,78,79,80,81,82,83,84,85] indicate the connectivity between food and I4. However, the following discussion is focused on the close connection between food agriculture and I4 from a socio-economic perspective.

Many such studies have been reported in the literature [35,71]. Hidayatno et al. [71] found that financial benefit increases I4 adoption in Indonesia’s food and beverage sector because an FSC utilizing an I4 innovation will determine the economy’s management capabilities. Kumar et al. [38] found 12 CE-related barriers to I4 implementation in SFSC. Cause–effect analysis and obstacle prominence evaluation were performed using Rough-DEMATEL. Managers, practitioners, and planners can benefit from knowing and overcoming the study’s findings.

Akyazi et al. [63] offered an industry-driven proactive plan for the food sector’s digital transformation. To achieve this purpose, they established the essential competencies and abilities needed for each food business’s professional profile. To achieve this, they established an automated database of current and prospective careers, skills, and talents. This database might guide the industry through I4’s revisions [63]. Academics and politicians believe that the I4FA Nexus supports the ecosystem’s societal growth. Kayikci et al. [41] established a blockchain-enabled FSC architecture, covering prospects and current barriers, based on a thorough literature analysis and semi-structured case interviews from emerging economies. They examined whether blockchain technology can solve FAI challenges, including traceability, trust, and accountability. Their work paved the way for future academics to address technological and human difficulties in the I4 age to lessen food business challenges. They gave instances of blockchain technology in I4, prompting more research and warning of the potential risks. The I4FA Nexus may cover and advance the ecosystem’s social (trust) progress.

Enarevba et al. [51] investigated combining Lean Six Sigma with I4 technologies in sub-Saharan Africa to decrease pre- and post-harvest food waste. The UN predicts a 33% worldwide population increase by 2050 and a 99% increase in sub-Saharan Africa. These expected trends will raise food security concerns, with sub-Saharan Africa facing the greatest demand growth. This I4FA Nexus covers the ecosystem’s socio-economic development. This I4 strategy is ideal for the Barranquilla food business since it meets logistics needs like FSC transparency and integrity management.

#### 4.1.3. Many Countries Have Started I4 in Their Food Agricultural Industries

**Figure 9:**
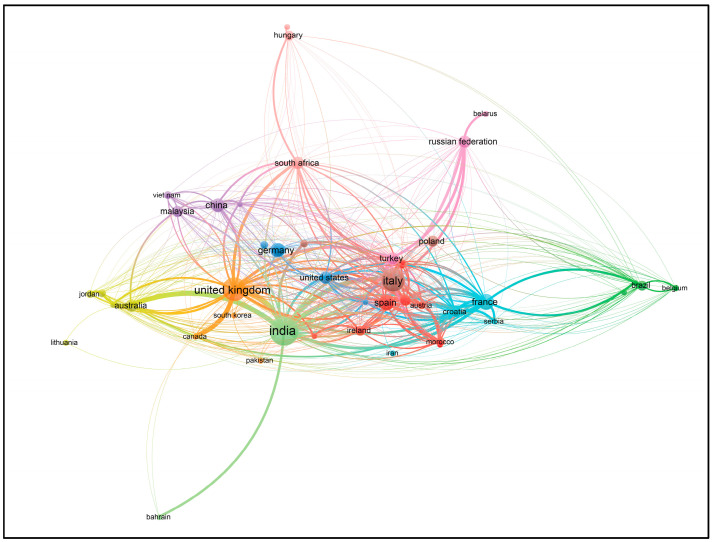
A bibliometric analysis of research created a visualization of the paper network, confirming the main themes of research, based on 49 countries with 11 clusters. The literature is based on the Scopus database; the keywords were ‘I4.0’ or ‘Industry 4.0’ and ‘Food’ and had to appear in the article title; 111 papers were found. The papers ranged from 2016 to 2024 and 2019 to 2023.

**Figure 10:**
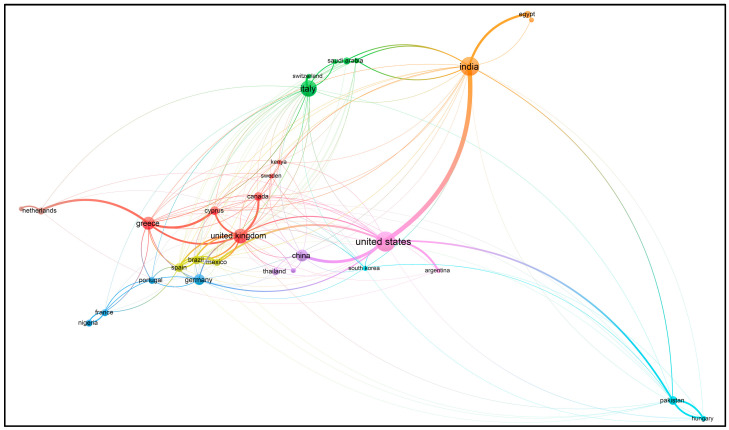
A bibliometric analysis of research created a visualization of the paper network, confirming the main themes of research, based on 36 countries with 9 clusters. The literature is based on the Scopus database on the topics of ‘sustainability (or sustainable) precision agriculture’, which had to appear in the article title; 82 papers were found. The papers ranged from 1995 to 2023 and 2002 to 2023.

Those countries that have started implementing I4 elements into their food agricultural production (in the phases from planting to marketing) included India [13,16,17,31], Russia [12,64], the UK [67,87], Italy [74], Australia [28,57], Indonesia [71], the European Union (EU) [79], Malaysia [88], Poland [42], Spain [44], Poland and Israel [19], Moldova [89], China [27], and the United Arab Emirates (UAE) [85] (Figure 9 and Figure 10).

Using VOSviewer software (version 1.6.20), the visualizations of the paper network-based data by clustering patterns confirmed the main themes of research based on countries; these visualizations are presented in Figure 9 and Figure 10.

##### Italy

Boccia et al. [74] recognized the potential of new technologies for food firms and their sustainability and management. The concept of the I4FA Nexus is thought to cover and support the ecosystem’s social development. In this Italian case, the function of I4 was to advance assistance towards the flexible chaining of the boards in worldwide frameworks and to perceive the possibilities of new advances for food organizations, their manageability, and their executives [74]. Romanello and Veglio [35] investigated the causes and effects of adopting I4 technologies in the context of an Italian food processing business. Their study emphasized the factors that influence and provide obstacles to the adoption of various I4 technologies. This I4FA Nexus is thought to encompass and contribute to the ecosystem’s social advancement. The effects of PA on nitrogen management were examined by Marinello et al. [109], who considered a 52 ha experimental site at a private farm in a typical Po Valley field in northeastern Italy. Using sustainable PA, they identified the crucial plantation area of corn (*Zea mays*), which can aid in defining the corrective measures that should be taken to lessen and minimize the effects of agriculture on the environment.

##### India

The I4 and circular economy (CE) adoption hurdles in the Indian agriculture FSC have also been highlighted by Kumar et al. [13]. They stated substantial barriers to implementing the I4-CE model, including a lack of government backing and incentives, regulations, and procedures. They reported that the stakeholders in the FSC will benefit from the research findings as they plan the strategic deployment of I4-CE. Arora [16] assessed the areas of I4 applicability to the Indian agricultural industry and how considerable advantages may be given to the farmers. Using the Delphi approach, the list of these digital use cases was then improved and prioritized while considering the use cases’ economic importance to Indian farmers and the simplicity of their implementation. A framework for cyber–physical agricultural systems (CPASs), which intelligently integrates CPS, IoT, CC, and big data with agricultural systems, was suggested by Sharma et al. [17]. CPAS may be used to increase productivity and leverage agricultural supply networks. The IoT-based global agricultural production management and control system, according to the I4 idea, was introduced by Szewczyk et al. [19].

Chatterjee et al. [31] studied how food and beverage firms will be affected by digital transformation in the employment of I4 technology in India’s post-COVID-19 era. As part of their path towards digital transformation in the post-COVID-19 situation, they reported that there was a substantial market for the food and beverage industries employing I4 technology.

##### The UK

The US and Europe have certain cultural commonalities and some cultural distinctions. For European farmers, one issue is the safeguarding of data as they are sent across platforms. Farmers in the US are less critical of this. The direct impacts of I4 technological capabilities (I4TC) and FSC integration on the efficiency of the sustainable agriculture FSC were studied by Sharma et al. [9]. Through the research of UK-based food and beverage producers, Kobnick et al. [67] have shown how deploying I4 activities is mostly tactical and hence divorced from the enterprises’ business models. They found that businesses must constantly develop their business models to apply I4. This I4FA Nexus is thought to cover and support the ecosystem’s social (manufacturing) advancement.

The digitalization of assembly through I4 operations, according to Koebnick and McFarlane [87], will impact all businesses, including the food and beverage industry. The analysis of UK-based food and beverage companies used in this article demonstrates that the usage of I4 exercises is typically strategic and, as a result, detached from the organizations’ action plans. This results from a lack of critical thinking about how I4 will affect their entire organization and about the prevalence of proficiency-arranged corporate societies.

##### Russia

The investigation of I4 principles was the focus of a study by Filatov et al. [64] to boost the competitiveness of the Russian Federation’s food and processing sector. The new revolution’s major challenge is less related to the technology than it is to the knowledge and training required to employ it. The extra benefits of multidisciplinary research and development are overlooked since the development of the new industrial revolution’s separate components is unpredictable. The social environment will alter significantly when production enters a new phase [64]. According to this I4FA Nexus, the ecosystem’s socio-economic development is covered. Aleksandrov et al. [12] analyzed digital transformations in agro-industrial complexes and identified the potential and risks for long-term socio-economic growth. They considered business cases for the effective digitization of agriculture by evaluating the economic impacts of digital technologies.

##### Australia

Ali and Aboelmaged [28] examined the perceived motivations and challenges associated with adopting FSC I4 in the food and beverage sector through interviews with top managers from the Australian beverage and FSC. They found that the key drivers for implementing FSC I4 are decreasing consumer needs, supply–demand misalignment, cost optimization, and the threat of legal penalties. They presented a fresh approach to qualitative data analysis that advances the field of FSC management’s methodology. This I4FA Nexus is thought to cover and contribute to the ecosystem’s socio-economic development (more job possibilities). Ali et al. [57] used a sample of 302 replies from senior managers in the Australian food processing sector as the basis for empirical testing. They discovered that the leading causes of FSC disruptions are supply–demand mismatches, process risks, and transportation risks. They alerted management to the adverse effects of FSC interruptions and the need for I4 technology to overcome the challenges. According to this I4FA Nexus, the ecosystem’s socio-economic development is covered.

##### Other Countries

Hidayatno et al. [71] conceptualized a systemic connection structure that could highlight the interactions between the policies and important factors influencing the adoption of technology 4.0 in the Indonesian food and beverage sector. Ushada et al. [33] simulated the trust-based decision-making process used by Indonesian small and medium-sized enterprise (SME) groups while adopting I4, namely ergonomic machines and e-commerce technologies. They used the Java, Sumatera, and Nusa Tenggara groups to develop the best trust and decision-making approaches. Ichsan et al. [72] showed the situation of the food and beverage manufacturing industry in Indonesia and the framework for implementing digital transformation in the direction of I4. According to this I4FA Nexus, the ecosystem’s socio-economic development is covered.

Oltra-Mestre et al. [44] studied the impact of I4 as a set of enabling technologies related to the core process innovation practice and product innovation of the FAI. They offered case studies of two Spanish businesses that processed fresh foods and competed in the meat, fruit, and vegetable industries, which are two significant industrial subsectors. This offered a framework for understanding how I4 technologies help researchers and management achieve competitive results by facilitating key innovation processes. According to this I4FA Nexus, the ecosystem’s socio-economic development is covered.

Pilinkien et al. [79] created a case study of the EU food sector by simulating several logistic network scenarios. They designed a competitiveness strategy based on the I4 idea and the lean philosophy. They demonstrated that a sustainable FSC, with minimal management costs and the visibility of the entire food chain, can be accomplished by deploying a logistic cluster in the EU and employing the devised competitiveness strategy. This I4FA Nexus is thought to cover and support the ecosystem’s social development.

In Poland, Kafel et al. [42] examined the official information on I4 and the digitalization elements offered by the Polish food organizations in response to I4 operations. Microsoft Excel forms were used to create charts utilizing the retrieved data. The data were then examined using both quantitative and qualitative content analysis. They found that activities carried out by Polish food organizations listed on the stock market increasingly showed signs of I4 and digitalization. Because of this, the top management boards are more confident and more interested in modernizing their I4-based operations. The ecosystem’s sociological (food organizations) and economic (stock exchange) advancement is thought to be covered and contributed to by this I4FA Nexus.

To propel its socio-economic progress and to achieve high-income nation status, Malaysia quickly grasped the reception of I4, according to Bujang and Abu Bakar [88]. Food and agribusiness production were identified as key factors in achieving this. The company as a whole, as well as the Andalusian food sector in particular, must implement the method suggested by I4. According to Luque et al. [80], it should be seen as an unusual advancement opportunity for the area. It is expected that, along with other industries, the food and beverage sector will embrace flexible and individualized manufacturing techniques [80].

Using an IoT-based approach, the broad rural creation of the board and control, as being necessary to the I4 notion, was proposed by Szewczyk et al. [19]. The four levels of the proposed framework—choice assistance, information handling, information collecting and transmission, and sensors—will be tested in Poland and Israel. An effective and efficient information procurement layer is essential for activities to succeed in the nation’s territory. Perciun et al. [89] analyzed the idea of I4 in agriculture through the current national and international expertise in digital technologies in Moldova. To assess the technical, human, and financial feasibility of using digital technologies to simplify agricultural firms’ management and to assure the sustainable growth of the national economy, they examined the situation of the Moldovan agro-industrial sector. They used digital technology to identify viable areas for agricultural growth and to assess the potential impact of their adoption on the production cycle and on raising the quality and competitiveness of domestic farm goods.

In China, the research of Sun et al. [27] found that the IoT significantly improved the activities of the CE. The practices of the CE included circular design, green manufacturing, re-manufacturing, and recycling. These environmentally friendly business practices complemented the company’s efforts to improve its environmental performance while boosting its economic performance. In the UAE, Kurdi et al. [85] empirically evaluated the effects of FSC I4 and FSC risk on organizational performance in the food manufacturing sector. They concluded that for food manufacturing enterprises to be competitive, efficient, and productive, they should start and develop their transition to FSC I4.

Therefore, all of the above literature reviewed points related to the fact that deploying I4 in the FAI in many countries is ever-expanding, now and in the future.

### 4.2. Good Prospects for the I4 Implementation in Food Agricultural Production

There are good prospects for the I4 technologies in PA for food agricultural production; these may be considered by looking at the social, economic, and environmental benefits.

#### 4.2.1. Social Benefits

Based on the literature review, the significant social benefits were (a) increased occupational safety and workers’ health and (b) increased food quality, security, and safety.

##### Increased Occupational Safety and Workers’ Health

Many studies indicated that the use of I4 in PA has benefited the food and agricultural sectors in terms of increased occupational safety and workers’ health [1,2,25,27,29,44,46,56,152,180,181,182].

When employed as seed priming agents, nanoparticles increase the seed germination rate, which benefits the plant’s overall development. Using insecticides and fertilizers with nanocapsules has revolutionized agricultural and animal health without harming the environment. The application of nanotechnology can effectively integrate various agricultural practices with sustainable production. Despite the various potential advantages of nanotechnology, it is crucial to consider the environmental safety risks carefully. Nanotechnology enhances their performance and sufficiency by boosting viability and security and reducing social insurance costs [152].

The potential of I4 in agriculture was previously covered by Knoke et al. [2]. Agritech Business 4.0 was updated using I4 technology by Sivakumar et al. [1] in 2021. I4 technologies are aligned with Agritech Business 4.0’s core components, including crop management, soil management, pest and disease management, water conservation, protection of farmers’ health, increased productivity, food safety, and FSC and the bolstering of the ties between urban and rural areas.

The main reason why human food is connected to I4 is because I4 is expected to assist and complement the FSC, food security, and food sustainability. Due to the fast-paced corporate climate, technology improvements, client preferences, growing competitive pressure, globalization of FSC, and environmental disturbances, the globe is witnessing technological disruptions. Digitalization initiatives have been increasing in the agri-food sector [29]. They must adopt new technology to ensure efficient and effective administration of their responsibilities. Although I4 technologies can provide chances for process innovation, how they affect innovation practices in the FAI needs more research output [44], which has been heavily challenged by climate change and population expansion [56].

To create smart factories with a strong focus on sustainability, Jambrak et al. [46] highlighted the need to consider the implementation of smart sensors, artificial intelligence (AI), big data, and additive technologies with nonthermal technologies. SWOT analysis revealed the potential for energy savings during food processing, optimized overall environmental performance, reduced manufacturing costs, the production of eco-friendly goods, improved working conditions, and a greater degree of health and safety during food processing. Advanced thermal and nonthermal technologies can be sustainable methods that comply with the United Nations Sustainable Development Goals (UN-SDGs). According to this I4FA Nexus, the SEE development of the ecosystem is covered. According to Senturk et al. [182], they employed a variety of digital devices in our daily lives, and these changes have been rather drastic. The usage of these technologies in diverse applications has recently been investigated in the agricultural and food industries. They suggested using these technologies, particularly IoT-based systems, to address the industry’s longstanding issues with food safety, mycotoxin contamination, pesticide residues, and growing waste. Sun et al. [27] stated that the IoT significantly enhanced CE activities, practices, and policies. They also significantly enhanced green manufacturing, circular design, remanufacturing, and recycling practices. An improvement in environmental performance can significantly impact a company’s success. By integrating IoT-based I4 technology into CE practices, their [27] research offered the framework for contributing nations/companies to achieve economic and long-term sustainability goals simultaneously. This I4FA Nexus is thought to cover and contribute to the ecosystem’s advancement on the economic and environmental fronts.

To identify the existence of abnormalities in the operation of industrial systems, Tancredi et al. [25] presented a structured approach that combines digital twin models, machine learning algorithms, and I4’s IoT. The suggested remedy has been created to be implementable in manufacturing facilities and is not explicitly intended for I4 applications. They found that two of the three machine learning algorithms were shown to be sufficiently successful in forecasting abnormalities [25] and recommended their deployment for the boosting of worker safety at industrial facilities. This I4FA Nexus is thought to cover and contribute to societal advancement of the environment (and the employees’ safety).

##### Increased Food Quality, Security and Safety

Many studies indicated that using I4 in PA has benefitted the food and agricultural sectors by increasing food quality, security, and safety [22,98,116,154,161].

Yadav et al. [22] examined these important agriculture FSC technologies by considering five research axes: information system management, traceability and food safety, food waste, decision making and agribusiness control and monitoring, and other ad hoc applications. They proposed that integrating the technologies they had evaluated might be more beneficial for offering affordable solutions and enhancing sustainability in agriculture FSC. Additionally, blockchain has the potential to revolutionize how food security and safety are achieved. This I4FA Nexus is thought to aid in the ecosystem’s socio-economic development. Regarding product quality, environmental concerns, and the welfare of humans and cattle, Cox [98] examined the technological advancements that boost agricultural and livestock output worldwide. They examined the methods for obtaining, using, and disseminating the necessary information. These stages are associated with the PA idea, which generally applies to crop and livestock production.

Preserving and responsibly using arable land resources are essential to ensuring global food security. Soil resources are under tremendous strain due to competition for land use from urbanization and commercial land use [116]. Land erosion and desertification are already causing the world’s arable land to decline, and our attempts to guarantee commercial land availability are worsening the situation. In addition to ensuring that the land is used as efficiently as possible, PA can improve the possibility of the global agriculture sectors being restored. One way to think of integrated nutrient and pest management is as future-proof land and water conservation, along with zero tillage, organic farming, and vertical planting [116].

According to Zhang et al. [154], global food security is being threatened by climate change, population growth, conflicting needs for land to develop biofuels, and deteriorating soil quality. There are excellent prospects for sustainable food production because of the convergence of PA, where farmers use artificial intelligence and nanotechnology to react in real time to changes in crop development [154]. To optimize targeting, uptake, delivery, nutrient capture, and the long-term impacts on soil microbial communities, it is possible to design nanoscale agrochemicals that combine optimal and functionality profiles by combining current nutrient cycling and crop productivity models with nano informatics approaches [154].

Food markets have been more globalized in recent decades as a result of trade agreements that have reduced protectionist laws, according to Saeys and De Baerdemaeker [161]. While this has made a more fantastic range of food goods more accessible to customers at lower costs, governments, merchants, and consumers worldwide increasingly worry about the safety and quality of their food products. PA technology can assist producers in meeting good agricultural practice standards and can relieve them of the administrative burden associated with demonstrating compliance, in addition to giving governments, merchants, and consumers the information they need to ensure food quality and safety [161].

#### 4.2.2. Economic Benefits

Based on all the literature reviews, the major economic benefits were (a) the use of sensors (IoT) to reduce the costs of agricultural production; (b) the reduction in costs via the FSC; and (c) the reduction in the costs of food production using the green technology of I4.

##### Use of Sensors (IoT) to Reduce Costs of Agricultural Production

Many studies considered the use of sensors (IoT) to reduce the costs of agricultural production [14,15,97,99,128,135,144,146,149,150,155,162,164].

By approaching sustainable intensification in agriculture to strike a balance between environmental stewardship and agricultural yield, PA has grown in popularity. Improving agricultural output while reducing adverse environmental effects is the goal of sustainable intensification. Using cutting-edge technology like IoT, GPS, GIS, sensors, drones, and machine learning has made it possible to complement this. This technology allows farmers to cultivate their land more precisely and efficiently [99].

To enhance the information layer and communication processes in the I4 architecture, Manogaran et al. [14] developed an information scheduling and optimization framework. Through the use of this framework, process delay and stagnancy are reduced through the best possible scheduling and classification of agricultural information. A smart farm’s control flexibility is calculated using the latency and stagnancy towards the end of yields. The classification component sorts data based on processing and completion times using offloading to remove backlogs. Mukherjee et al. [15] addressed the impact of I4 on the agricultural FSC. I4 examined how the agriculture FSC may benefit, and it completed a thorough examination of the literature. The agriculture FSC industry is one of them. It also shows how I4 in FSC management for agriculture may be applied to boost productivity, customer satisfaction, and efficiency. Their study may help forecast the future interactions between I4 and agriculture FSC management, bringing I4 and Agriculture 4.0 together.

PA makes a significant contribution to sustainable agriculture [144]. Technological advancements were the foundation for the multidisciplinary conversation and the creation of these novel approaches. PA became conceivable with the global positioning system and the new sensor systems made available by information technology. Farm automation, site-specific farming, fleet management, and field robots are all made feasible by the applications of these technologies. This can be carried out by optimizing farm, plant, machine, and job management [144]. Spatial planning for agriculture growth can be aided by implementing web-based information systems, an essential component of IoT technology [146].

According to Patel et al. [162], the Indian agricultural sector has significant challenges in achieving food and environmental security in the new millennium, as indicated by the country’s rapidly growing population and diminishing production. In terms of improving the land’s carrying capacity sustainably, PA technologies may be the best choice [162]. The arrival of new ICT technologies within the broader IoT framework has made PA adoption possible [128]. The design of networks for PA can be supported by formal software engineering models and procedures, according to Bodei et al. [128].

The PA technique maximizes the production of high-quality crops by monitoring the environment and field conditions while reducing environmental pollution with little input (e.g., fertilizer, herbicides, and pesticides) [135]. However, a fundamental barrier to the widespread adoption of PA is still the absence of data; these data are a crucial aspect of the achievement of PA. Additionally, Kim and Lee thoroughly examined and described electrochemical sensors—such as those that track soil, plant development, and environmental factors [135].

To understand machine learning’s use in agriculture, Priya et al. [149] offered the fundamental idea of the technology as well as the systematic procedures. Sangeetha et al. [150] stated that nanoparticles are a potential medium for drug administration because of their ability to pass through this barrier with ease and without the need for outside assistance. So, by using genetic engineering, nanoparticles may be able to transfer biomolecules to plants. When fertilizers and pesticides are used carelessly, the environment is contaminated, and biodiversity is threatened [150].

In ornamental nursery production, over-fertilization is a widespread practice [155]. Fertilizer treatment estimations are often inaccurate since visual inspection is frequently utilized to assess plant nutrition levels. Two non-destructive sensors, Soil Plant Analysis Development (SPAD-502) and GreenSeekerTM, were investigated by Freidenreich et al. [155] to determine whether they were suitable for detecting the absorption of nutrients into plant tissue. As an efficient and non-destructive instrument for sustainable fertilizer management practices in the ornamental plant business, their technique might be used as a reference for nursery producers and landscaping staff.

According to Fountas et al. [97], who studied the methodologies and implementation of PA throughout the previous 25 years, the acceptance of technology and its impacts on crop management, the environment, and the sustainability of agricultural systems are all related. For each field at the site-specific level, the farm manager may obtain data on soil, yield spatial distribution, weather, crop scouting, remote sensing, and yield collecting methods. Enhancing productivity and profitability while mitigating environmental impacts will be possible with new sensors that identify anomalous responses in the soil or crops [97]. A state-of-the-art chemical sensor system was created to analyze Thai sustainable PA chemically at a reasonable cost for use in rural Thailand and other locations [164].

##### Reduction in Costs via the Food Supply Chain

Many studies indicated that using I4 in PA has benefitted the food and agricultural sectors in reducing costs via FSCs [22,24,26,28,36,48,50,52,55,56,66,74,75,122].

These FSCs and agricultural productions may be improved with I4 solutions, resulting in higher product quality, greater food output, and optimized operations, among other advantages. Using the production of and market for chicken as an example, the interconnections between FSC resilience, I4, and sustainability are examined [56]. The UN estimated global food losses and waste in 2011 at 1.3 billion tonnes annually [66].

The objective of Perez Perales et al. [75] was to classify these technologies according to the two following standards: the primary subjects to be addressed in each objective and the FSC participant where it is performed specify the environmental or social goal to be achieved [75]. They focused on technologies that address environmental and social sustainability because economic sustainability will rely on the particular characteristics of the company (an FSC using a certain I4 may be successful while others are not). The social evolution of the ecosystem is assumed to be covered by and supported by this I4FA Nexus.

Lopes et al. [55] offered a technique for using CE business models to solve losses and waste in the FSC. Initially, a comprehensive literature review was conducted to determine how CE is used at the cutting edge of the food waste industry. In terms of management contributions, they suggested deploying CE business models more widely to solve food losses and waste while accounting for the retail tier’s participation. This I4FA Nexus covers the socio-economic evolution of the ecosystem.

Mohajeri et al. [52] offered a model of the advantages of operations for the food reverse FSC by putting the I4 concept into practice. A device that recycles household waste was introduced as an example of the I4. Electric cars have also been considered for delivery and pickup by I4. Recyclable stations have defined the rate of progress. Many methods for recycling food waste using different technologies have been selected and assessed based on the I4 indicators. Food waste is sent to recycling stations, which are establishments maintained, operated, or used to purchase, sell, or store it before recycling it by using the appropriate machinery. The model’s several goals minimize the negative effects of environmental degradation and transportation costs while maximizing the benefits of recycling and consumer response. In this work, the whale optimization approach is applied. They provided a comprehensive reverse supply chain management method for food waste based on the I4. According to this I4FA Nexus, the ecosystem’s SEE evolution is covered.

I4 represents a group of CPSs. It supports the idea of “smart factories”, wherein machinery is given online access, linked to a production process monitoring system, and empowered to make decisions independently. This framework showcases the emerging innovations that are happening all around the world, especially in Europe. It might lead all enterprises to more competitive outcomes and to intriguing results in the years to come. In this context, there seems to be substantial room for growth, even for the most innovative component of FSC management—the service sector [74]. Nevertheless, this potential needs to be adequately exploited and backed by targeted investments. The FAI is undergoing significant change due to I4’s increasing digitalization. Smart technology is altering the dynamics of the FAI, requiring increased automation. Thanks to the new automation phase, the sector may now enjoy streamlined, dependable, and efficient processes, services, and products, but it also requires new professional capabilities from its personnel. It is critical to identify the near-term skill requirements for the sector to close the skill gaps between the labor force and the industry demands [48].

I4 is required across the FSC to handle the rising global demand for food products and the concerns about food security and safety [22]. Green technologies have drawn significant interest in many food applications, even though I4 technologies are changing various production and consumption sectors, including the food and agriculture industries [24]. Poor food quality and safety lead to foodborne illnesses and costly food crises, eroding consumer confidence, and reducing the effectiveness of cold food chains. I4-related modifications to food traceability systems, using automatic identification and sensor technologies instead of manual paper-based record-keeping, can improve data transfer and self-monitoring to reduce issues related to food quality. Before selecting a technology to meet a certain need, it is important to assess its performance with regard to many considerations [36]. The two main issues that industrial firms grapple with are adopting I4 technologies, which automate and boost plant productivity, and evaluating more environmentally friendly items and processes [26]. FSC 4.0-related research on the factors influencing investment in these technologies is still in its early stages despite a notable increase in knowledge related to I4 technologies [28]. Companies that produce food and packaging will need to transition from a linear to a CE by implementing policies that can increase the sustainability of their operations and products from an environmental, social, and economic perspective. Thus, food companies must reinvent themselves to stay competitive in the market by using innovative methods and tools to boost the efficiency and output of their establishments [50].

Regarding employment, turnover, and added value, the food and beverage industry was once again recognized as the largest manufacturing sector in the European Union in 2017, according to Bucci et al. [122]. Nonetheless, most businesses are SMEs, which exhibit a sluggish pace of innovation and PA technology adoption. With the arrival of the digital era, agri-food SMEs are finding new ways to apply technology advancements throughout the FSC—from farm to fork—to boost their competitiveness. Their [122] study, which addresses the state of the art, affirms that technology applications in food production are essential for guaranteeing sustainable farming systems.

##### Reduction in Costs of Food Production Using Green Technology of Industry 4.0

Many studies have indicated that the deployment of I4 can reduce the costs of food production [26,79,141].

Concerns about food security, climate change, and population expansion are causing agriculture to undergo a digital revolution. Information technology affects agriculture in ways that lower costs while increasing productivity and sustainability. To help with the field identification of pests, plant diseases, and inadequate plant nutrition, PA uses IoT, deep learning, predictive analytics, and AI-based technologies. The following are the goals of the study: (1) To examine the function of smart technologies and how they affect the sustainability of PA; (2) to consider the usual use of deep learning and IoT data analytics in PA; and (3) to look at the obstacles to the adoption of sustainable PA. For an in-depth study, IoT devices gather data and send them to data analytics and deep learning [141]. According to Micheni et al. [141], the data help farmers to manage crop diversity, phenotypes and selection, crop performance, soil quality, pH level, irrigation, and the amount of fertilizer applied. Their analysis focused on important PA success elements and common application domains. Technology adoption is influenced by cost, privacy, safety, and legal and technological concerns. The research will be useful to government agencies, academic institutions, individual farmers, and agricultural authorities.

In the European Union, SME ranchers provide 49.6% of the food consumed in the region. Although the administration is committed to paying for the excess, the Common Agricultural Policy was designed to guarantee a market for SME ranchers. Food is deemed trash when it has passed its expiration date or is supplied to other company sectors at substantially reduced pricing. This problem prompted the authors to construct a contextual study of the food business in the European Union by showcasing various well-calculated organizational scenarios and implementing an intensity system based on the I4 concept and lean methodology [79].

Technological, environmental, economic, and social considerations were considered in Stefanini and Vignali’s [26] assessment of automated guided vehicles (AGV) as an I4 application. The systems’ environmental and economic impacts were compared using life cycle costing and the life cycle assessment approach, which was performed using the SimaPro 9.1 application. Social concerns concerning the workers’ working circumstances were considered in the 4.0 scenario. The evaluation’s conclusions can benefit companies considering using AGVs for material handling and can contribute to the corpus of scientific knowledge. The question of whether adopting AGVs will lead to more sustainable end logistics processes in the food company was addressed with their foundation. According to this I4FA Nexus, the SEE development of the ecosystem is considered.

#### 4.2.3. Environmental Benefits

PA can help manage food agricultural production inputs in an environmentally friendly way. Based on all the literature reviews [90,91,92,93,94,95,96,97,98,99,100,101,102,103,104,105,106,107,108,109,110,111,112,113,114,115,116,117,118,119,120,121,122,123,124,125,126,127,128,129,130,131,132,133,134,135,136,137,138,139,140,141,142,143,144,145,146,147,148,149,150,151,152,153,154,155,156,157,158,159,160,161,162,163,164], the major environmental benefits were (a) the reduction in chemical leaching, avoiding excessive fertilizer application; (b) the increase in energy efficiency; and (c) the reduction in food wastes (recycling) using green technology.

##### Reduction in Chemical Leaching Avoiding Excessive Fertilizer Application

Based on the present literature review, many studies have focused on PA using a more environmentally friendly fertilization application [90,91,92,94,99,102,109,110,111,117,121,139].

PA enhances field-level management for sustainable food production. Sustainable farm production includes the alignment of agricultural practices to soil fertility, crop demands, and environmental circumstances [121]. PA aims to increase farm profits by (1) efficient resource management through the variable-rate application of nutrients, agrochemicals, and water; (2) reducing crop yield losses during harvesting; (3) minimizing environmental risks (e.g., greenhouse gas emissions and nutrient leaching); and (4) optimizing farming input footprints. Site-specific agricultural inputs are needed to maximize farm earnings and safeguard the environment with PA technology [121]. PA is involved in food security, environmental preservation, sustainable resource utilization, and economic advantages. Yield monitoring, remote sensing, and efficient fertilizer, water, and pesticide delivery to crops are covered. Thus, food production and resource efficiency may be maximized without waste or environmental damage from excessive fertilizer or pesticide use [94].

PA aids the environment by targeting inputs to decrease losses from excess applications, nutrient imbalances, weed escapes, insect damage, etc. Reduced pesticide resistance is another benefit. Few publications have analyzed the measured environmental variables directly, such as by leaching with soil sensors. Most of the calculated indirect environmental benefits are derived by assessing chemical loading reduction [90]. PA technologies for food security and sustainability are vital resources that review PA research across disciplines. It also addresses innovative tools and approaches to improve system implementation. Engineering and computer science are used in PA research to enhance crop health, irrigation, and fertilizer use [102].

Farm management today must satisfy ecological, economic, and social needs. Due to various legislation, farmers must achieve sustainability and environmental protection standards. More commonly, they must record, archive, and validate data [117]. Comprehensive planning modules allow graphical planning and execution of PA activities, including cultivation, cropping, fertilization, pest management, and harvesting. Fertilizer application, including PA, illustrates planning, execution, and graphical and tabular (database) assessment [117]. Van Evert et al. [93] examined how conventional PA practices boost profitability and sustainability. They calculated each scenario’s output, input, and environmental values. This allowed us to compute profit and social profit, which is revenues minus conventional expenses minus external production costs. Sustainability may be measured by social profit. PA boosts olive sustainability and potato profitability and sustainability. Nath [99] envisioned sustainable intensification and examined PA’s role. PA practices, such as precision irrigation, fertilizers, pest and disease control, and animal farming, are highlighted in this review. Thus, technology innovation, sustainable farming, data analytics, and legislative interventions will shape sustainable PA.

Peerlinck and Sheppard [102] optimized winter wheat crop yield output to boost farmers’ production. Optimization might lead to poor sustainability if too much fertilizer is applied or the farming equipment is overworked. Therefore, they included sustainability targets that directly address these issues. A novel multi-objective factored evolutionary algorithm solves multi-objective optimization using overlapping subpopulations. Their results showed that multi-objective optimization with overlapping subpopulations improves objective space exploration. PA is used in olive orchards (*Olea europaea* L.) to manage agronomic variability and give plants the correct input quantity without loss [111]. Roma et al. [111] developed a GIS platform employing GEOBIA algorithms to create prescription maps for variable rate (VRT) nitrogen fertilizer treatment in olive orchards.

Dubos et al. [139] compared the optimal N and K rates advised by each approach in adult oil palm using long-term fertilization experiments. Leaf analyses (LA) yielded modest rates relative to nutritional balance. LA showed each block’s prospective yield clearly. They concluded that this perfectible technology was more environmentally friendly and did not reduce yields or soil mineral reserves.

##### Increase in Energy Efficiency

Based on the present literature review, many studies have focused on PA by increasing energy efficiency during food agricultural production [113,123,126,145,147].

Agricultural irrigation has attracted attention to the boosting of agricultural production and the conservation of water. Traditional irrigation uses water and electricity to schedule irrigation [123]. A fuzzy-based intelligent irrigation scheduling system employing a low-cost wireless sensor network was proposed by Jamroen et al. [123]. A cost study verified the irrigation scheduling system’s economic viability. Energy-intensive cereal-based farming techniques in South Asia’s Indo-Gangetic Plains distort agricultural income and the environment [126].

Achour et al. [147] reviewed recent greenhouse technology used in hardware design, environmental monitoring, dynamics modelling, microclimate control, energy optimization, green energy integration, and storage system implementation. Renewable energies like solar and geothermal have become extensively adopted as ecologically benign alternatives, making greenhouse energy self-sufficient and able to exchange electricity with the grid. The Agri.q for PA can map, monitor, manipulate, and collect small soil and crop samples in unstructured agricultural environments due to its modular articulated mechanical structure and specific sensors and tools, according to Botta and Cavallone [145]. Sustainable 5G PA is hindered by sensor node (SN) battery capacity [113]. Chien et al. [113] proposed a system for charging SNs and gathering sensory data using unmanned aerial vehicles to overcome this challenge.

##### Reduction in Food Waste (Recycling) Using Green Technology

Based on the present literature review, many studies have reported a reduction in food waste (recycling) using green technology during food agricultural production [67,80,183,184,185]. This is also well indicated in Figure S1, where the industrial revolutions complemented the agricultural revolutions, from Industry 1.0 to I4 [11].

The digitization of manufacturing through I4 initiatives will influence several industries, including the food and beverage industry [67]. The integration of PA with smart grid technology has been proposed by Odara et al. [157] as a potential strategy for augmenting the capacity of sustainable energy supply. Agriculture is a significant burden due to its many chores, including irrigation, crop harvesting, and processing. Integrating such practices holds promise for the enhancement of agricultural systems by mitigating input expenses, especially those associated with waste management. Moreover, the use of carbon-neutral fuels might potentially have good environmental outcomes.

The predicted adoption of I4 is expected to enhance production capacity, increase output value, and assist the government in attaining its economic objectives [71]. The food sector holds a prominent role in the economy of Andalusia, owing to its significance, advantages, and potential. Consequently, it poses a substantial challenge within the region’s economic framework. Implementing the framework proposed by I4 is of utmost importance for the whole sector and specifically for the FAI in Andalusia. It should be regarded as a significant opportunity for businesses to progress. It is expected that, as with other sectors, the food and beverage industry will emerge as a frontrunner in using adaptable and personalized manufacturing techniques [80].

**Figure 11:**
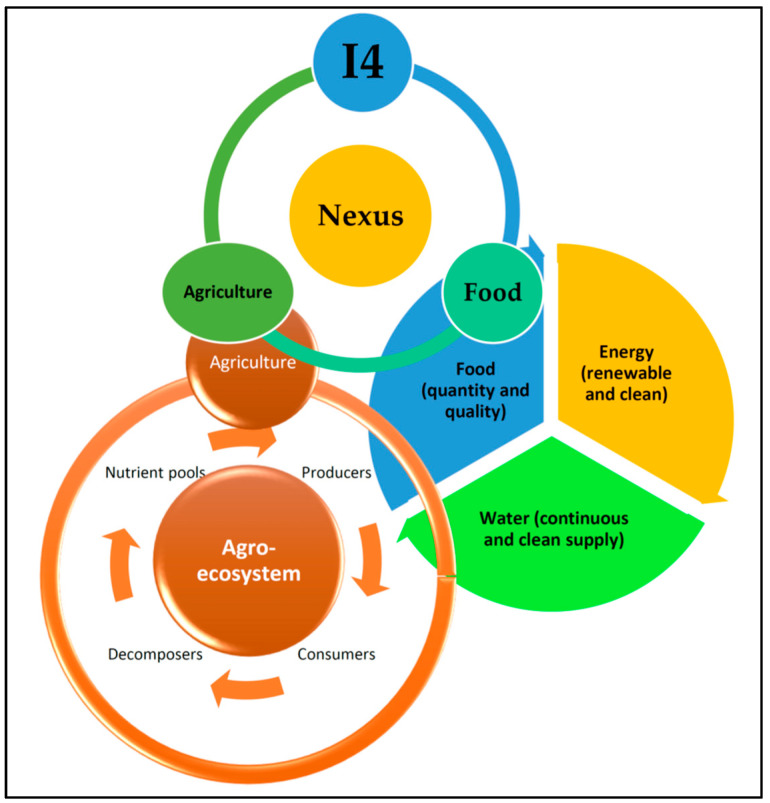
An idealistic conceptual relationship between Industry 4.0 (I4)—Food-Agriculture Nexus and agroecosystem (with three related to United Nations Sustainable Development Goals), with foods (in abundance in terms of quantity and quality, as indicated by deep green color) in the society (present and future) when environmental stress (climate change) factor is taken into account.

Logically, there are always reasons for the connectivity between the I4FA Nexus and the agroecosystem (Figure 11). The primary reason behind this is the increasing pattern in electricity production worldwide [183] (Figure S2). The per capita (kilocalorie) supply from all foods per day [184] (Figure S3) in the past few decades has shown a very positive increment, and this pattern is expected to continue in the foreseeable future. Overall, the per capita calorie supply has steadily risen worldwide. However, these patterns differ in different parts of the world. In recent decades, the caloric supply in Asia and Africa has increased significantly. For the previous several decades, there has been convergence in the worldwide trends in caloric supply due to the greater growth in the world’s poorer regions. However, there was an inverse pattern for the renewable freshwater resources (RFRs) per capita [185] (Figure S4). The overall amount of renewable flows and the population density determine per capita RFRs. Per capita, renewable withdrawals will decrease if RFRs diminish, which can often happen in nations with significant yearly rainfall variation, such as during monsoon seasons. Similarly, if total RFRs stay the same, the per capita levels may decrease if a nation’s population increases. Population growth is causing many countries’ per capita RFRs to decrease.

### 4.3. The Challenges of Sustainable Agricultural Food Industry in Adopting Industry 4.0

On the other hand, the I4 technology-driven nature combined with the relatively early phases of the I4 technologies life cycle implies and raises several concerns. Based on all the literature reviews, the major issues of the sustainable FAI in adopting I4 were (a) the question of preparedness, (b) lack of trust, (c) privacy issues, and (d) economic revenue uncertainties.

Feeding a rising population, ensuring farmers’ livelihoods, and conserving the environment are the three difficulties facing the world’s food systems, according to Brooks et al. [186]. The three challenges in the incorporation of a socio-economic environment must be faced simultaneously if lasting progress in any of them is to be achieved. Thus, this is crucial. Given the scope and complexity of these issues, policymakers may need to try out new techniques to create a set of answers that appeal to all stakeholders.

Food agriculture as a sustainable and non-substitutable resource has been well supported and should not become a debatable issue now and in the future. Several facts and figures in the past have justified this agricultural sustainability from the point of view of the ecological agroecosystem and, lastly, from a socio-economic perspective. A population predicted to increase depends on the global food system to feed its members with safe and nourishing food. In addition to additional mouths to feed, the demand for meat, fish, and dairy will increase as wages rise in emerging and developing nations [186]. The advancement of existing technology and fresh suggestions concerning switching from outdated methods to more effective ones for manufacturing nutrient-dense foods are presented [187]. To solve all of the above problems, the use of I4 in PA is recommended.

I4 may make managing agroecosystems easier in order to produce secure food and nutrition. Theoretically or fundamentally, agroecosystem management is essential to preserve ecological stability, social equality, economic viability, and cultural vitality [92]. Also, it is in tandem with the UN-SDGs, especially with regard to Zero Hunger (Goal No. 2), Responsible Consumption and Production (Goal No. 12), Life Below Water (Goal No. 14), and Life on Land (Goal No. 15).

The impacts of an increased amount of atmospheric CO_2_ emissions that could potentially adversely affect our future agricultural output and food quality were considered. The agroecosystem now places a fresh emphasis on the hot subject of climate change. These risks can alter the environment abruptly or gradually, hurting biotic processes and deteriorating abiotic circumstances. Although the I4FA Nexus complementing the agroecosystem is an almost perfect approach, determining how many hurdles and obstacles first need to be overcome by the less privileged countries or industries is again a never-ending discussion. Below are some of the potential challenges of incorporating I4 in PA.

#### 4.3.1. Social Predicaments

These involve changes in human behavior and mindsets during the paradigm shift. The social issues included in the following discussion are (a) the question of the preparedness of small industries (social adaptations), (b) lack of trust, and (c) privacy issues [188,189,190].

##### The Question of Preparedness

This mainly involves industrial modifications. I4 manageability with economic practices has received a significant financial commitment. Implementation will have SEE effects. A significant financial investment, time for adaptation, especially in less-privileged nations or businesses, and a shift from the human ecological paradigm are needed.

SME businesses often employ century-old machinery. Konur et al. [59] presented a unique case study of switching a traditional food producer to I4. The article describes their development and transition challenges. They showed smart production control CPSs. The system’s novel data collection, information extraction, and intelligent monitoring services had increased productivity and consistency while lowering operational expenses. Similar food production and SME industries can benefit from the approach and learning. To avoid mass technological unemployment, a social ecosystem for seamless technology adoption with social design is needed [64]. This I4FA Nexus covers ecosystem socio-economic development.

Farm production is moving towards IoT-based smart systems with smart items as the world becomes digital. This trend is expected to accelerate as AI-powered devices and smart technologies grow more widespread. Smart objects detect conditions and respond intelligently without human intervention. Real-time agricultural field monitoring saves money, manages resources, and informs choices. The IoT, a key I4 enabler, has enabled innovative agriculture technology for cost reductions and output increases and improved big data analytics for future choices. However, limited-resource agriculture struggles to modify production to suit present needs [7]. I4 might modernize smart farming by improving productivity and reducing human intervention. This smart paradigm automates planting and output yield using innovative methods. Farm systems are improved by adding the I4 paradigm to intelligent computer and communication technology [14].

Pérez Perales et al. [16] focused on natural and socially sustaining developments. Manageability and economic practices are crucial in most organizations’ internationally flexible supply networks [68,191]. Most companies utilize this to manage production, services, and corporate social responsibility. Most companies employ manageability to meet client requirements in the present supportable social consciousness, which includes food production. Ojo et al. [68,77] linked I4 to food-producing FSC standards. Thus, economic practices for I4 deployment in the agricultural food business may make managing it difficult.

These business models have created new FAI labor skill needs [63]. Precision pest control is being introduced in the developed world using artificial neural network-based machine learning (pheromone-based visual traps for insect identification) and electric nose technology-based automatic machines or sensing devices for hotspot (infestation area) identification. These technologies are expensive and sophisticated. Therefore, resource-poor farmers are reluctant to use them [175]. Thus, the efficacy of using I4 smart technology for insect pest control and precise pest management is still debated [175]. According to Furstenau et al. [172], the scientific community focuses on economic and environmental conditions while overlooking social issues. Thus, many discussions and debates have always concerned I4 and sustainability research challenges, perspectives, and concepts. Facchini et al. [23] examined the competitiveness risks and opportunities by determining the “readiness degree” of agri-food enterprises to employ smart technology. They used smart technologies to measure the company’s economic, social, and sustainable competitiveness. This I4FA Nexus may help the ecosystem’s socio-economic growth.

De Carolis et al. [188] accurately anticipated that digital technology will drive manufacturing transformation in I4. In practice, such technologies allow firms to find ways to turn increased complexity into long-term competitiveness and profitable growth. However, the practice still affects industrial implementation. According to Cotrino et al. [189], I4 technologies like the IoT, virtual reality, and CC are changing company structures in manufacturing and small SMEs. A literature analysis found that most large companies have investment strategies, some of which are reviewed in this paper. The major projections show that the major enterprises’ I4 investments exceed the SMEs’ yearly revenues, making it difficult for SMEs to obtain these technologies. The study found two gaps: the newest literature study does not explore I4’s practical use in SMEs, and there are no I4 implementation roadmaps for SMEs. SME finance cannot pick the finest technology, design the best strategy, and pay for extensive consultancy help. They showed SMEs how to access I4 technology with inexpensive investments.

Hizam-Hanafiah et al. [28] discovered 30 I4-ready models with 158 dimensions. The prevalence of technology among these six most prominent qualities suggests more research on I4 preparation. Mechanized farming displaced indigenous farming during the first two industrial revolutions, and PA is new. Industrial farming increases productivity, but some challenges have become increasingly important. I4 is expected to accelerate the fourth agricultural revolution [11]. Climate change, resource limitations, changing customer demands, and rigorous regulations are continually on stakeholders’ minds in the FAI, which utilizes many resources. The food business has implemented I4. Improving transparency through AR experiences is a key focus. Although I4 technologies are used more in the FAI, AR is still underutilized [53]. I4, the current industrial revolution, has transformed the dynamics of the industry as a whole, causing the food business to evolve quickly.

This digital revolution is real, but which digital technology will benefit each business field is uncertain [5]. Baierle et al. [5] analyzed the adoption of digital technologies in several industrial sectors to see which of them may boost agricultural system performance. They analyzed industrial sectors to create a digital transformation framework to boost FAI competitiveness in Agriculture 4.0. The food sector frequently uses only one digital technology. Therefore, they showed the need for concurrent and joint investments in the other technologies addressed in this research. Public policy must stimulate the FAI’s digital technology development [5]. Arora et al. [6] analyzed the use of these technologies in agriculture and created a priority ranking based on how effectively they overcome these difficulties. Two steps were taken in their research. First, I.4.0 technologies and agricultural FSC bottlenecks were identified. A discussion follows on the proposed framework, which blends data envelopment analysis with analytical hierarchy. They found that agricultural technology can improve FSC management. Their research prioritized options based on final weights. This ranking system can help farmers and the government choose the best technologies to automate the agricultural FSC.

Naqvi et al. [7] converted conventional agriculture into IoT-enabled smart systems to address quality issues. According to Bernhard et al. [8], some agricultural regions need improvement. Bernhardt et al. [10] investigated whether there were techniques and whether these structures were suitable for agriculture. I4’s approaches help agriculture, they reported. Agriculture has different structures; thus, they must be changed. Liu et al. [11] investigated industrial agriculture’s contemporary situation and the lessons learned from industrialized agricultural production patterns, processes, and the agri-FSC. They focused on the critical scientific issues and agricultural applications of these technologies.

##### Lack of Trust

Societies may only encourage more sustainable farming systems by developing policies that incorporate SEE concerns [180,181,182,183,184,185,186,187,188,189,190,191,192]. When advising SME businesses in the food and beverage industry to implement I4, policymakers consider trust a key factor. According to their knowledge, familiarity, agreement, and preferences, the SMEs’ degree of trust in executing I4 is described as their belief in using the right technology for I4. Several Kansei terms, or factors relating to human thinking, are included in the complicated concept of trust [58]. I4 is the most prominent example of a technical breakthrough that may help businesses and entrepreneurs address these difficulties in such a scenario [20]. Digital technologies also play a similar role in the PA sector. Therefore, although I4 is a future paradigm shift in the FAI, its deployment faces many socio-economic consequences that need time for adaptations and mindset adjustments.

Ushada et al. [34] aimed to deploy I4 in food and beverage industry SMEs by modelling group preference decision making. The travelling salesman problem-based decision-making process was modelled using an ant colony optimization approach. They showed that equipment and tools were the most popular choices for I4 implementation. When choosing the first characteristic, adaptability was the top choice. They anticipated that the high confidence level in group choices would support I4’s sustainability. The method adds to several already-existing theoretical frameworks for decision making based on group preferences and can help the management of SME’s to implement I4.

Ushada et al. [58] utilized artificial neural network modelling to simulate SMEs’ confidence in implementing I4. They found that the result was a categorization of trust as “overtrust”, “trust”, or “distrust”. They showed that education, knowledge, familiarity, benefits, preference ranking, and linguistic components all impacted SMEs’ levels of trust.

##### Privacy Issues

Prasad et al. [3] analyzed the significance of numerous applications, including smart agriculture, smart cities, smart healthcare, and smart medicine, as well as their features, security problems, and privacy concerns. Along with future study topics and breadth, frameworks for reducing the effect of security and privacy problems are also highlighted. An AI-based smart farming protocol was presented by Mahajan et al. [4] since AI techniques are crucial for enhancing the performance of I4 standards. Using clustering and routing methods, they created the lightweight clustering protocol for I4-enabled PA.

A broad framework is developed in Bigliardi’s [49] thorough literature evaluation of the use of the I4 paradigm in the food business. A basic review of green and I4 technologies from a food viewpoint was presented by Hassoun et al. [24]. The UN-SDGs and I4 enablers (such as artificial intelligence, big data, smart sensors, robots, blockchain, and the IoT) will be connected to green food technologies (such as green preservation, processing, extraction, and analysis). These technologies promise to promote ecological and digital changes in food systems that will benefit society, the economy, and the environment. While the use of digital technologies and other I4 technology advancements in the FAI is still in its infancy, various green technologies have already offered creative solutions for significant changes in the food system.

#### 4.3.2. Economic Revenue Uncertainties

This is especially due to the cost-intensive nature and difficulties involved in estimating full financial benefits and economic effectiveness, as indicated by some published studies [13,20,21,59,190].

The relationship between the domains of I4 and PA was considered by Trivelli et al. [20]; they examined the most prevalent technologies employed in each area to identify similar trends and technological overlaps. A method combining manual and automated analysis was created to do this. They discovered a lexicon of 324 words related to PA technologies, a graph outlining the relationships between the technologies, and a depiction of the major technology clusters observed. To provide thoughts and concerns for the future, Zambon et al. [21] analyzed retraces of the stages of the industrial and agricultural revolutions that have occurred up to the present. To enable the effective application of I4 principles, they examined the unique difficulties faced by agriculture throughout the FSC.

Agribusiness organizations have started implementing technology to create an FSC that is more sophisticated, customer-focused, and sustainable. Even if the adoption of linked new technologies and the CE concept poses many difficulties, they have already shown their utility in the industrial sector in achieving a sustainability goal [13]. The interaction between people, machines, and electronics in today’s industries is considered to be a smart ecosystem that is necessary for the efficient production of goods. I4 is a group of technologies that serve as enablers for such intelligent ecosystems and enable the transformation of industrial processes. However, the need for modernization and automation at conventional factories makes it necessary to overcome several practical obstacles when adopting and implementing I4 [59].

#### 4.3.3. Environmental Impacts of Industry 4.0 in Food Agriculture

The implementation of Industry 4.0 in food agriculture can have negative impacts on the environment. One negative impact is the increased use of resources, such as energy and water, due to the integration of advanced technologies. This increased resource consumption can contribute to the environmental degradation and stress already caused by limited resources. Additionally, digitizing and automating agricultural processes can lead to biodiversity loss.

By replacing manual labor with machines and robots, there is a potential decrease in the diversity of plant and animal species that traditionally coexist in agricultural ecosystems. Industry 4.0 and the digitization of agricultural processes can lead to a further intensification of production methods, which can negatively impact soil quality and cause water pollution and the excessive use of chemicals. Moreover, the reliance on digital technologies and connectivity in Industry 4.0 can also increase the vulnerability of food agriculture systems to cyber-attacks and data breaches. Furthermore, the increased reliance on technology and automation in Industry 4.0 can lead to the loss of traditional farming practices and local knowledge, potentially disrupting agricultural communities’ cultural and social fabric. While Industry 4.0 offers numerous benefits for the food agriculture industry, such as increased efficiency and productivity, it is crucial to carefully consider and address the potential adverse environmental impacts to ensure sustainable and responsible implementation. Implementing Industry 4.0 in food agriculture can have positive and negative environmental impacts. The digitization and automation of agricultural processes in Industry 4.0 can decrease biodiversity by replacing manual labor with machines and robots. This can disrupt the balance of plant and animal species in agricultural ecosystems. Additionally, the intensification of production methods in Industry 4.0 can negatively impact soil quality and cause water pollution and the excessive use of chemicals. Furthermore, the increased reliance on digital technologies and connectivity in Industry 4.0 can increase the vulnerability of food agriculture systems to cyber-attacks and data breaches.

### 4.4. The Knowledge Gaps for Future Studies

**Figure 12:**
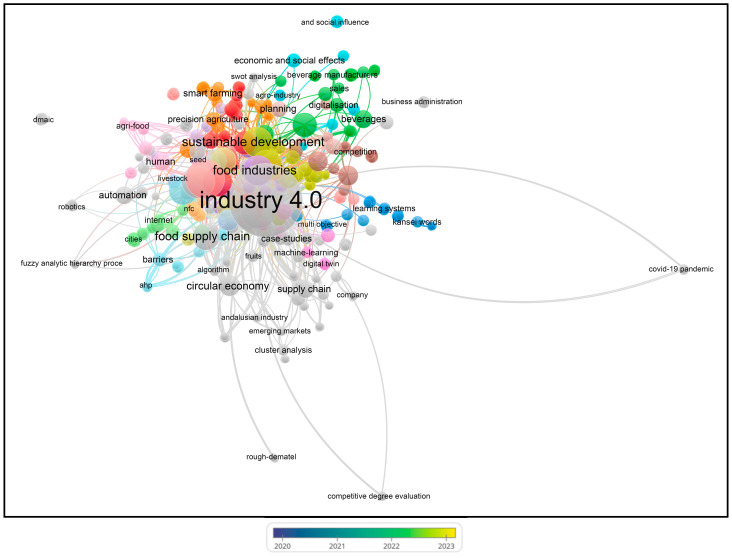

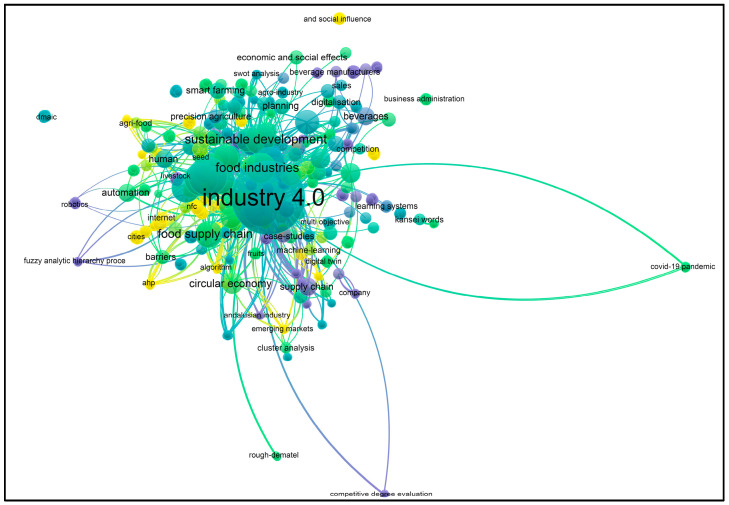
A bibliometric analysis of research themes on ‘Industry 4.0’ and ‘Food’. Top panel: visualization of the paper network confirming the main themes of research. Bottom panel: evolution of research trends between 2019 and 2023. The colors in the top panel indicate the themes of research that are being discussed in the papers while in the bottom panel the colors indicate the year of publication.

This gives a holistic overview of the past research based on the keywords’ co-occurrences with ‘Industry 4.0’ and ‘Food’ (Figure 12). The analysis reveals a discernible prominence reflecting three principal domains of investigation, namely three significant clusters that can be identified based on visualization in Figure 12 (top panel): (a) sustainable development, (b) food industries and the food supply chain, (c) the circular economy and the supply chain. Finally, recent studies focused on smart cities, emerging markets, agri-food competition, and machine learning (Figure 12 bottom panel).

**Figure 13:**
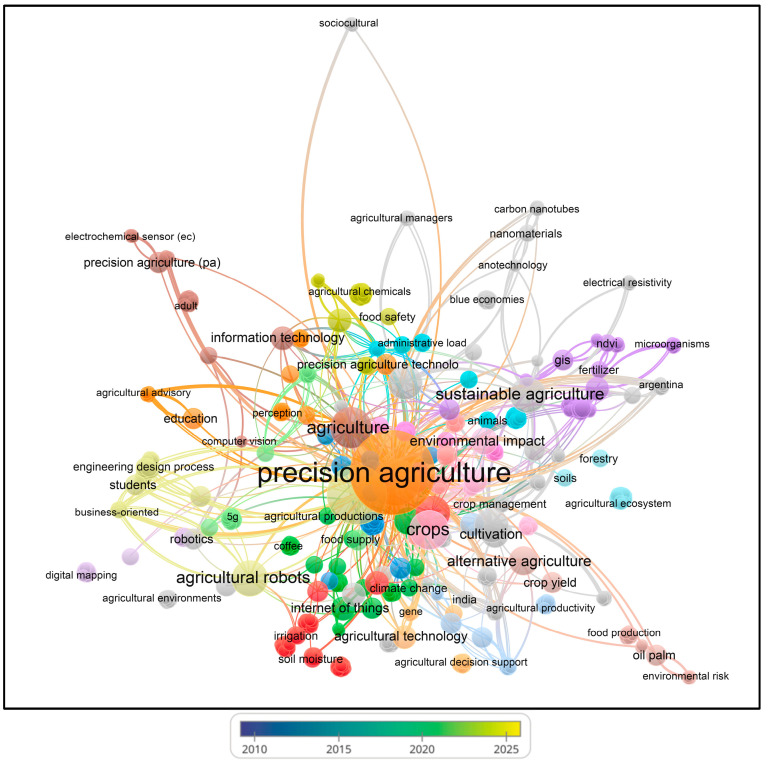

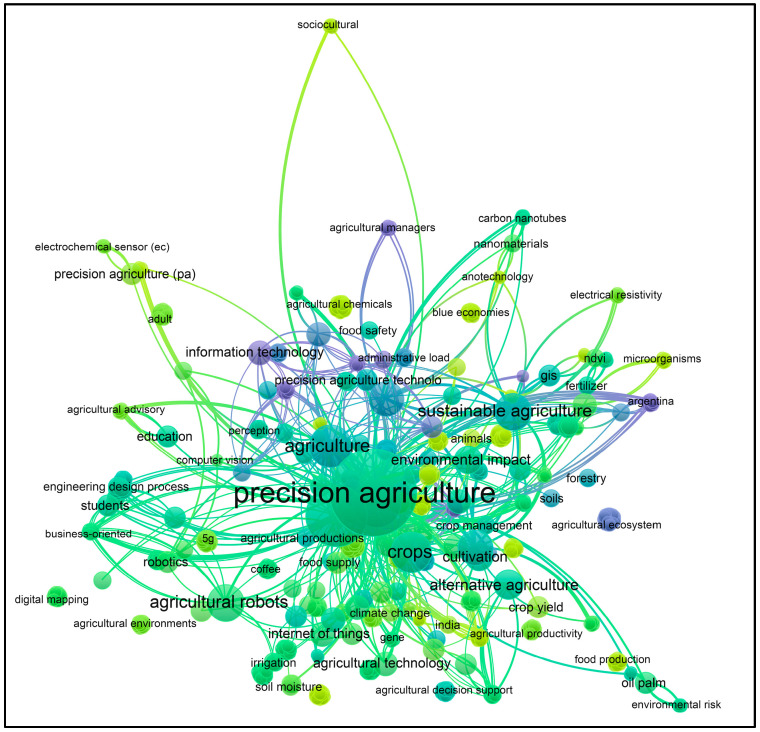
A bibliometric analysis of research themes on the ‘sustainability (or sustainable) precision agriculture’. Top panel: visualization of the paper network confirming the main themes of research. Bottom panel: evolution of research trends between 2019 and 2023. The colors in the top panel indicate the themes of research that are being discussed in the papers while in the bottom panel the colors indicate the year of publication.

Similarly, a holistic overview of the past research based on the keywords’ co-occurrences with ‘sustainability (or sustainable) precision agriculture’ is presented in Figure 13. The analysis reveals a discernible prominence reflecting three principal domains of investigation, namely three major clusters that can be identified based on visualization in Figure 13 (top panel): (i) agriculture, crops, agricultural robots, and sustainable agriculture; (ii) environmental impact, alternative agriculture, and the Internet of Things; and (iii) information technology, precision agriculture technology, cultivation, crop yield, and agriculture production. Recent studies focus on food production, climate change, crop yield, blue economy, agricultural chemicals, and animals (Figure 13 bottom panel).

Interestingly, in both visualizations in Figure 12 and Figure 13, there have been no studies on implementing ESG to effectively manage I4’s deployment in the agricultural food sectors. Therefore, the ESG is highly under-studied. This is a visible knowledge gap between EGS and I4 implementation in the FAI. It is now receiving more and more of the attention from the agricultural food industries in their annual governance reports. Future studies should focus on ESG implementation as the solution for effective manageability of I4’s deployment in the agricultural food sectors.

#### 4.4.1. Importance of ESG in Precision Agriculture

Environmental, social, and governance (ESG) principles have been applied to the agricultural food industries’ businesses [192,193,194,195,196,197,198,199]. The data on ESG are important for several reasons as they provide valuable insights into a company’s sustainability, responsible practices, and long-term performance [194,195,196,197,198,199].

ESG considerations are crucial in agriculture. They strengthen local communities, encourage moral labor, reduce environmental damage, and improve governance [192]. ESG principles may help agriculture preserve the environment, progress society, and sustain the economy. ESG considerations may help agribusinesses attract ethical investors, fulfil customer demand for sustainable and ethical products, and reduce resource shortage and climate change concerns [193,194]. ESG integration improves risk management, sustainability, stakeholder confidence, and customer demand for ethical and environmentally friendly products [195]. Sustainable and ethical practices improve social inclusion, economic stability, FSC resilience, and environmental protection [196,197,198,199].

This study is relevant because agricultural enterprises require a new management culture that considers global environmental threats to humanity. At a time when Russia’s green (responsible) finance sector is just starting to grow, agriculture is one of the most attractive areas for capital investment to sustain development, preserve biocapacity, and lead the globe. The issues are defined. Due to its conservative management and state regulatory monopoly, the agriculture business is unattractive for venture capital and green finance from banks, which hinders innovation and sustainable growth [192]. Agriculture and forestry are key businesses. As ESG grows, stakeholders are more interested in its impact on agricultural and forestry company performance [193].

Dorashka et al. [192] systematically studied the global green financing of agribusiness enterprises and the Russian ESG financing market to develop specific proposals for the involvement of agribusiness enterprises and financial institutions in financing sustainable development projects as an objective necessity for life on Earth. Zeng and Jiang [193] used two-stage least squares to examine the theoretical and empirical implications of ESG for corporate performance in 156 listed agricultural and forestry enterprises. They stated that (1) ESG and corporate performances are strongly correlated and that higher ESG ratings improve corporate performance; (2) social and governance performances are better at encouraging business performance growth than environmental performance; and (3) there are no discernible differences between listed firms in forestry and agriculture with regard to how ESG affects corporate performance. They also advised listed companies to promote green growth. Their findings helped listed agriculture and forestry firms to boost ESG performance and corporate success.

Buallay [194] examined how sustainability reporting affects agricultural operations and financial and market performances. According to their statistics, ESG has no substantial relationship with operational, financial, or market performance. Governance transparency positively affects market performance when each ESG element is separately regressed against performance, which is surprising. Hrebicek et al. [195] examined an organization’s environmental, social, economic, and governance (ESG) variables in examining corporate sustainability reporting trends in the agricultural and food processing industries. The relationship between environmental and sustainability metrics and corporate sustainability reporting needs to be revised [195].

Business, environmental, economic, and social data are recorded, standardized, registered, and collated into key performance indicators [196]. The organization can acquire and incorporate these data in the corporate sustainability or environmental report if such requests arise [197,198]. The combined achievement of ESG performance metrics would measure business success in various economic activities. Sustainability performance is sometimes characterized as environmental, social, and economic/financial performance, ignoring governance [199]. The ESG and the indicators do not focus on the agriculture sector, which affects many food processing sustainability issues and all linkages in the FSC. The ESG in the Food Processing Sector Supplement includes food sector efforts to promote the environmental, social, and economic sustainability of food production chains, including agriculture [195]. The present literature on I4FA [200,201,202,203,204,205,206,207,208,209,210,211,212,213,214,215,216,217,218,219,220,221,222,223,224,225,226] supports this.

#### 4.4.2. Environmental Factors in Agribusiness ESG

**Figure 14:**
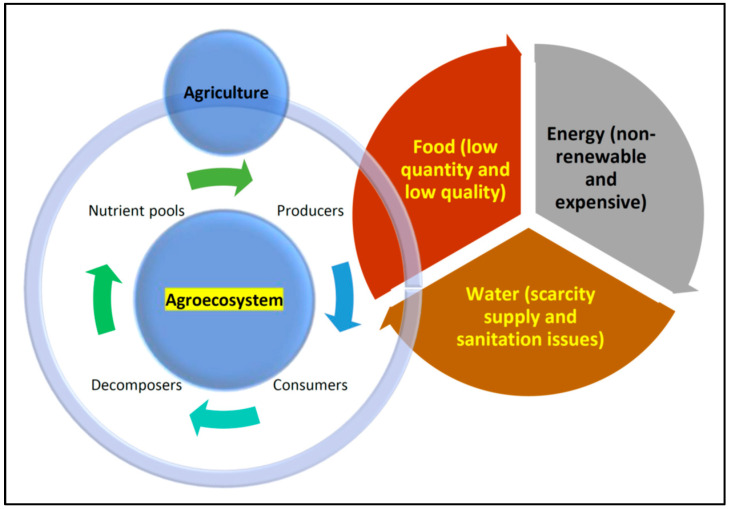
A conceptual relationship between agroecosystems, where food items are deficient in quantity and quality (as indicated by the deep blue color) in today’s and future societies under the presence and impacts of environmental stresses (climate change) factor.

This is because environmental stresses are drivers of food supply deficiency. Figure 14 shows the conceptual relationships between the agroecosystem and three related UN-SDGs, where food items are deficient in quantity and quality in today’s and future societies under the presence and impacts of environmental stresses (climate change) factors. Therefore, the connectivity between agriculture and I4 stems from the need for food sustainability and sustainable FSC [200,201,202,203,204,205,206,207,208,209,210,211,212,213,214,215,216,217].

Pollution, climate change, unsustainable land use [165,166,167], unsustainable farming practices, and overexploitation of resources [168] are well-known factors that stress our global agroecosystem in the efforts to balance production demand with population growth. Agroecosystem stress reduction strategies are critically required. Switching to green food production is smart. Agroecosystem ecotoxicologists are studying climate change aspects to decrease human influence and preserve natural ecosystems. Agroecosystem management helps to meet the UN-SDGs, including Zero Hunger, Life on Land, Responsible Consumption and Production, and Life Below Water. More specifically, the UN-SDGs’ success depends on agroecosystem sustainability.

I4 technology is growing in popularity, but how it may be conceptually integrated to supplement the agroecosystems’ renewable resources remains a knowledge gap and a global conversation. This review paper seeks to connect and discuss how idealistic conceptual relationships between the I4FA Nexus and the agroecosystem can be logically connected and married. This food (in abundance and quality) in society (present and future) is under environmental climate change stress.

Population increase, climate change, food waste, and pandemics have hampered global food security [39]. Understanding how to preserve the agroecosystem to keep an ever-supplied food quantity under the iconic CE of I4 applications will be crucial to developing a sustainable FSC [57]. This complete approach to sustainable production and consumption with limited and contaminated natural resources has made the CE idea popular globally [43].

A healthy PA agroecosystem with I4 will provide high-quality, sustainable food (Figure S1). Due to ESG implementation, sustainable land and water management, natural resource preservation, and biodiversity preservation were achieved. Agriculture needs biodiversity preservation to survive. It involves safeguarding natural ecosystems, native species, and biodiversity-friendly farming practices and avoiding pesticide and chemical fertilizer applications [192,193,194,195,196,197,198,199].

ESG implementation provided climate change mitigation and adaptation plans, as described above. Agribusinesses must reduce greenhouse gas emissions and adapt. To minimize fossil fuel use, agro-forestry, PA, soil carbon absorption, and renewable energy can be used [192,193,194,195,196,197,198,199].

**Figure 15:**
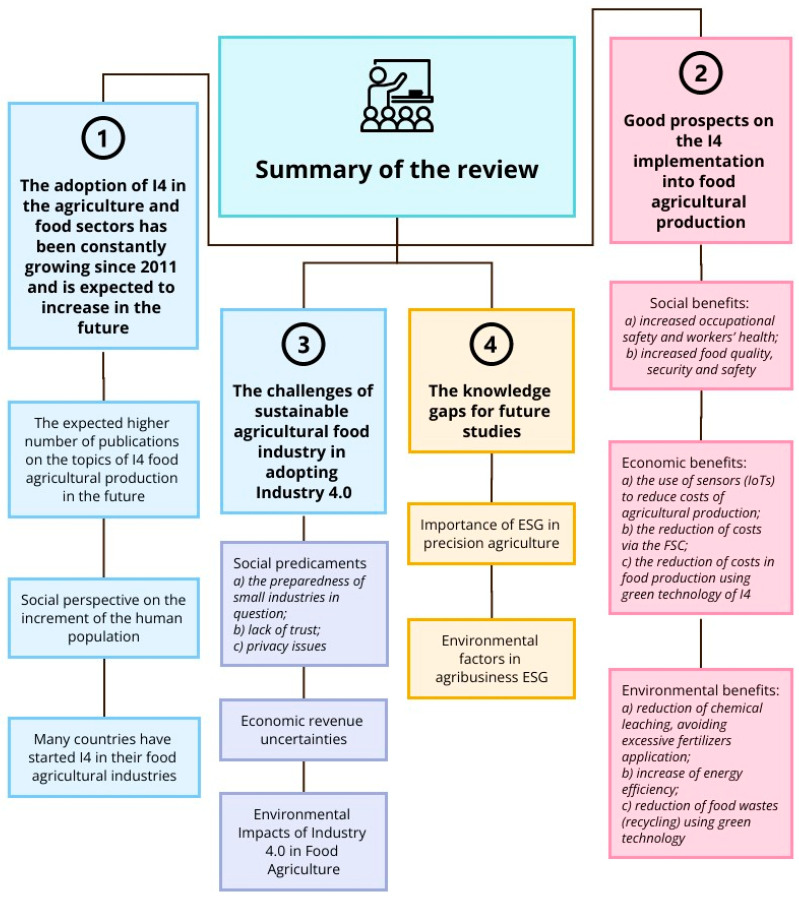
Overall review outcomes and the knowledge gaps from the present review study.

Thus, ESG improves risk management and sustainability in agriculture. Agricultural risk management could be improved using ESG to identify and mitigate resource shortages, climate change, and regulatory changes. Morality and adaptation to changing conditions promote long-term viability. Overall, one of the proposed ways of understanding the knowledge gaps in the food agroecosystem is ESG implementation (Figure 15).

## 5. Concluding Remarks

The above analysis throws light on four fascinating issues to consider. To begin, the use of I4 technology in the agriculture and food industries is expected to continue to increase both now and in the future. Second, there are good prospects for the I4 implementation in food agricultural production. This paper discussed the social benefits, including increased occupational safety, workers’ health, and increased food quality, security, and safety. The economic benefits were the use of sensors (IoT) to reduce the costs of agricultural production, the reduction in costs via the FSC, and the reduction in the costs of food production using the green technology of I4. The environmental benefits included the reduction in chemical leaching, the avoidance of excessive fertilizer application, the increase in energy efficiency, and the reduction in food wastes (recycling) using green technology.

Third, there are always challenges facing the sustainable FAI in adopting I4. This paper discussed the challenges related to the preparedness of small industries (social adaptations), lack of trust, privacy issues, economic revenue uncertainties, and some environmental impacts. Even though I4 is anticipated to be a paradigm shift in the future of food agriculture, its implementation will have several SEE impacts. These impacts will require time for adaptation, particularly in industries or countries with fewer resources, as well as a significant financial commitment and a shift in thinking away from a human ecology frame of mind.

Fourth, the knowledge gaps for future studies were identified as the ESG to be proposed as the solution for effectively managing I4’s deployment in the agricultural food sectors. For agriculture to be considered sustainable with regard to ESG, it is essential that all aspects of sustainability, including social, economic, and environmental sustainability, cooperate. In addition, there is always the need to provide reasons for the relationship between the I4FA Nexus and the agroecosystem.

From this review, the concept of I4FA might be brought into the real world with an open-minded conversation platform with ESG-minded leaders that could help complement sustainable agroecosystems worldwide.

## Supplementary Materials

The following supporting information can be downloaded at: https://www.mdpi.com/article/10.3390/foods13010150/s1, Table S1: A literature search on keywords ‘I4.0’ and ‘Food’ found in different regions or countries, Table S2: The names of journals and their numbers (found 111 papers) published based on Scopus database with keywords ‘Industry 4.0’ and ‘Food’, ranging from 2016 to 2024, Table S3: The names of journals and their numbers (found 82 papers) published based on Scopus database with keywords ‘‘sustainability (or sustainable) precision agriculture’, ranging from 2015 to 2023, Figure S1: Diagram of development roadmap of industrial revolutions and agricultural revolutions. Diagram cited from Liu et al. [11], Figure S2: Electricity production (TWh) worldwide from 1990 to 2022 [183], Figure S3: Per capita kilocalorie supply from all foods per day from 1961 to 2020 from different regions and worldwide [184], Figure S4: Renewable freshwater resources per capita of different regions in the world from 1961 to 2019 [185].
