# Supplementary material for: A Conceptual Model Relationship between Industry 4.0—Food-Agriculture Nexus and Agroecosystem: A Literature Review and Knowledge Gaps
Source: Foods. 2024 Jan 1;13(1):150. doi: 10.3390/foods13010150 (PMC10778930; doi:10.3390/foods13010150)
Supplement: Supplementary file 1 [file foods-13-00150-s001.zip › foods-2756345-supplementary.pdf]

foods

Review

A conceptual model relationship between Industry 4.0 – Agriculture - Food Nexus and Agroecosystem: A literature review

Chee Kong Yap [1,*] and Khalid Awadh Al-Mutairi [2]

[1] Department of Biology, Faculty of Science, Universiti Putra Malaysia, 43400 UPM Serdang, Selangor, Malaysia
[2] Department of Biology, Faculty of Science, University of Tabuk, Tabuk P.O. Box 741, Saudi Arabia; kmutairi@ut.edu.sa
* Correspondence: yapchee@upm.edu.my

Table S1. A literature search on keywords 'I4.0' and 'Food' found in different regions or countries.

No.		Country	Reference
1	I4.0 and Food Supply Chain	Italy	[74]
2	I4.0 in Food and Beverage Manufacturer	Indonesia	[17, 33, 71-72]
3	I4.0 competitiveness strategy: Sustainable food supply chain	European Union	[79]
4	Universal Data Acquisition Module PIAP-UDAM for I4.0 Application in Agriculture	Poland and Israel	[19]
5	Digital business model innovation in the food and beverage industry	UK	[87]
6	A data-driven approach to galvanize Malaysia's agro-food sector development through Agriculture 4.0	Malaysia	[88]
7	Application of I4.0 to the Andalusian food industry	Spain	[80]
8.	I.4.0 as an incentive to increase the competitiveness of the food and processing industry	Russian Federation	[64]
9.	I4.0 technologies could act as enablers of competitive outcomes and the core innovation processes.	Spain	[86]
11.	To identify promising areas of agricultural development based on digital technologies, and to evaluate the possible effect of their implementation on the production cycle and increasing the quality and competitiveness of domestic farm products.	Moldova	[89]
12.	Incorporating IoT-based I4.0 technology into CE practices to attain economic and long-term sustainability goals.	China	[27]
13.	Businesses in making better-informed decisions about adopting supply chain I4.0. The food manufacturing companies should initiate and advance their transition to supply chain I4.0 for them to be competitive, effective, and productive.	UAE	[85]
14.	Focused on food and beverage companies that are using I4.0 technologies or are in the process of digital transformation. Developing the unique conceptual model is an important contribution to the body of knowledge.	India	[31]
15	Highlighting the managers' concern to the negative impact of supply chain disruptions and the significance of I4.0 technologies in dealing with adversities.	Australia	[57]

Table S2. The name of journals and their numbers (found 111 papers) published based on Scopus database with keywords 'Industry 4.0' and 'Food', ranged from 2016 and 2024.

No	Name of journal or proceedings	Number of paper
1	2016 Manufacturing and Industrial Engineering Symposium: Innovative Applications for Industry, MIES 2016	1
2	2018 IEEE 16th Student Conference on Research and Development, SCOReD 2018	1
3	2018 IEEE International Conference on Technology Management, Operations and Decisions, ICTMOD 2018	1
4	2021 IEEE International Conference on Technology and Entrepreneurship, ICTE 2021	1
5	2022 ASABE Annual International Meeting	1
6	2022 IEEE 12th International Conference on RFID Technology and Applications, RFID-TA 2022	1
7	ACM International Conference Proceeding Series	3
8	Advanced Series in Management	1
9	Advances in Intelligent Systems and Computing	2
10	Advances in Mechanical Engineering	1
11	Agronomy	1
12	Amfiteatru Economic	1
13	Annals of Operations Research	1
14	Applied Sciences (Switzerland)	1
15	British Food Journal	4
16	Business Strategy and the Environment	1
17	Cleaner Logistics and Supply Chain	1
18	Communications in Computer and Information Science	1
19	Computer Aided Chemical Engineering	1
20	Computers and Industrial Engineering	1
21	Creativity and Innovation Management	1
22	Critical Reviews in Food Science and Nutrition	1
23	Current Nutrition and Food Science	1
24	Cybernetics and Systems	1
25	Deutsche Lebensmittel-Rundschau	3
26	E3S Web of Conferences	2
27	Energies	1
28	Engineering Proceedings	2
29	Environmental Footprints and Eco-Design of Products and Processes	1
30	European Journal of Innovation Management	1
31	Food Engineering Innovations Across the Food Supply Chain	1
32	Food Manufacture	2
33	Food Research International	1
34	Foods	1
35	Frontiers in Psychology	1

36	IEEE Access	1
37	IEEE Internet of Things Journal	1
38	IEEE Journal of Radio Frequency Identification	1
39	IEEE Transactions on Engineering Management	2
40	IEEE Transactions on Industrial Informatics	1
41	IFAC-PapersOnLine	1
42	IFIP Advances in Information and Communication Technology	1
43	Impacts of Climate Change on Agriculture and Aquaculture	1
44	Information (Switzerland)	1
45	Innovation Strategies in the Food Industry: Tools for Implementation, Second Edition	1
46	International Journal of Advanced Manufacturing Technology	1
47	International Journal of Integrated Supply Management	1
48	International Journal of Knowledge-Based and Intelligent Engineering Systems	1
49	International Journal of Mechatronics and Manufacturing Systems	1
50	International Journal of Production Research	1
51	International Journal of Productivity and Performance Management	2
52	Internet of Things: Technological Advances and New Applications	1
53	IOP Conference Series: Materials Science and Engineering	1
54	Journal of Cleaner Production	2
55	Journal of Engineering and Technological Sciences	1
56	Lecture Notes in Mechanical Engineering	2
57	Lecture Notes in Networks and Systems	2
58	Lecture Notes of the Institute for Computer Sciences, Social-Informatics and Telecommunications Engineering, LNICST	1
59	Microprocessors and Microsystems	1
60	Mobile Information Systems	1
61	Neural Computing and Applications	1
62	Procedia CIRP	1
63	Procedia Computer Science	1
64	Procedia Manufacturing	1
65	Proceedings of the 2022 7th International Conference on Computing, Communication and Security, ICCCS 2022 and 2022 4th International Conference on Big Data and Computational Intelligence, ICBDCI 2022	1
66	Proceedings of the 30th International Conference of the International Association for Management of Technology, IAMOT 2021 - MOT for the World of the Future	1
67	Proceedings of the Summer School Francesco Turco	2
68	Processes	2
69	Production Planning and Control	2
70	Quality Assurance and Safety of Crops and Foods	1
71	RISTI - Revista Iberica de Sistemas e Tecnologias de Informacao	1
72	Rivista di Studi sulla Sostenibilita	2
73	Schweizerische Zeitschrift fur Forstwesen	1

74	Sensors	2
75	Sensors (Switzerland)	1
76	Serbian Journal of Management	1
77	Smart Agricultural Technology	1
78	Smart Food Industry: The Blockchain for Sustainable Engineering: Volume I - Fundamentals, Technologies, and Management	1
79	Statisztikai Szemle	1
80	Sustainability (Switzerland)	4
81	Sustainable Food Processing and Engineering Challenges	1
82	Technology Analysis and Strategic Management	1
83	Trends and Issues in Interdisciplinary Behavior and Social Science - Proceedings of the 5th International Congress on Interdisciplinary Behavior and Social Science, ICIBSOS 2016	1
84	Trends in Food Science and Technology	2
85	Uncertain Supply Chain Management	1
86	VDI Berichte	1
	Total	111

Table S3. The name of journals and their numbers (found 82 papers) published based on Scopus database with keywords ''sustainability (or sustainable) precision agriculture', ranged from 2015 and 2023.

No.	Name of journal or proceedings	Number of papers
1	2022 IST-Africa Conference, IST-Africa 2022	1
2	Advanced Sustainable Systems	1
3	Agriculture (Switzerland)	1
4	Agriculture, Livestock Production and Aquaculture: Advances for Smallholder Farming Systems: Volume 2	1
5	Agronomy	2
6	Agronomy Journal	1
7	American Journal of Alternative Agriculture	1
8	ASEE Annual Conference and Exposition, Conference Proceedings	2
9	Atti della Societa Toscana di Scienze Naturali, Memorie Serie B	1
10	Biochemical Systematics and Ecology	1
11	Biogenic Nano-Particles and their Use in Agro-ecosystems	1
12	Biomaterials Advances	1
13	Biosystems Engineering	1
14	Bodenkultur	1
15	CEUR Workshop Proceedings	1
16	Cleaner Environmental Systems	1
17	Computer Communications	1
18	Computers and Electronics in Agriculture	1
19	Conference Proceeding - 5th International Conference, TAE 2013: Trends in Agricultural Engineering 2013	1
20	Decision Support Systems	1
21	Energy	1
22	Engineering for Rural Development	1
23	Entrepreneurship and Sustainability Issues	1
24	Frontiers in Chemistry	1
25	Frontiers in Plant Science	2
26	GAIA - Ecological Perspectives for Science and Society	1
27	Global Climate Change: Resilient and Smart Agriculture	1
28	Global Environmental Politics	1
29	Heliyon	1
30	IEEE Access	2
31	IEEE Micro	1
32	IEEE Transactions on Industrial Informatics	1
33	Indian Phytopathology	1
34	Indonesian Journal of Electrical Engineering and Computer Science	1
35	International Journal of Ecology and Environmental Sciences	1
36	International Journal of Sustainable Agricultural Management and Informatics	1

37	International Water and Irrigation	1
38	IOP Conference Series: Earth and Environmental Science	1
39	Journal of Agricultural and Food Chemistry	1
40	Journal of Cleaner Production	1
41	Journal of Commercial Biotechnology	1
42	Lecture Notes in Civil Engineering	1
43	Lecture Notes in Computer Science (including subseries Lecture Notes in Artificial Intelligence and Lecture Notes in Bioinformatics)	1
44	Mechanical Engineering	1
45	Mechanisms and Machine Science	1
46	Nano-enabled Sustainable and Precision Agriculture	1
47	Nanotechnology for Agriculture: Advances for Sustainable Agriculture	1
48	Nanotechnology for Environmental Engineering	1
49	Nature Plants	1
50	OCL - Oilseeds and fats, Crops and Lipids	1
51	Open Life Sciences	1
52	Plants	2
53	Precision Agriculture	4
54	Precision Agriculture for Sustainability and Environmental Protection	2
55	Precision Agriculture Technologies for Food Security and Sustainability	1
56	Precision Agriculture: Evolution, Insights and Emerging Trends	2
57	Proceedings - 2015 IEEE International Conference on Technological Innovations in ICT for Agriculture and Rural Development, TIAR 2015	1
58	Proceedings - 2023 International Conference on Control, Artificial Intelligence, Robotics and Optimization, ICCAIRO 2023	1
59	Quality - Access to Success	1
60	Renewable and Sustainable Energy Reviews	1
61	Research Anthology on Strategies for Achieving Agricultural Sustainability	1
62	Scientia Horticulturae	1
63	Site-Specific Management for Agricultural Systems	1
64	Soil and Tillage Research	1
65	Springer Climate	1
66	Supply Chain Management for Sustainable Food Networks	1
67	Sustainability (Switzerland)	3
68	Sustainable Computing: Informatics and Systems	1
69	VDI Berichte	2
	Total	82

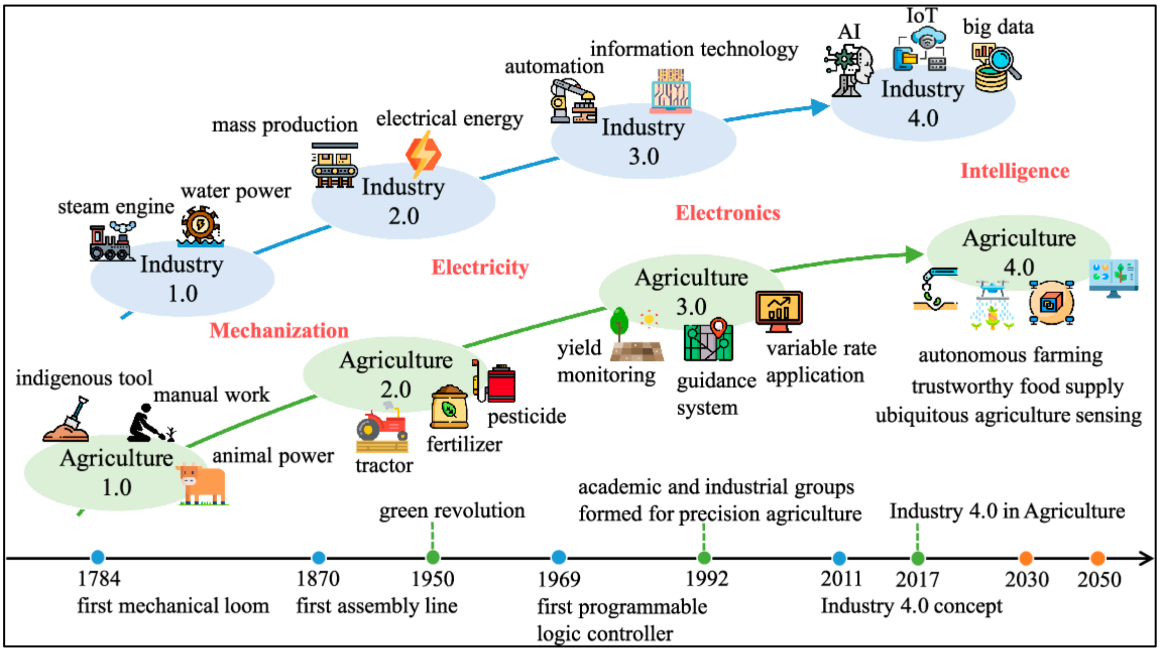

Figure S1: Diagram of development roadmap of industrial revolutions and agricultural revolutions. Diagram cited from Liu et al. [11].

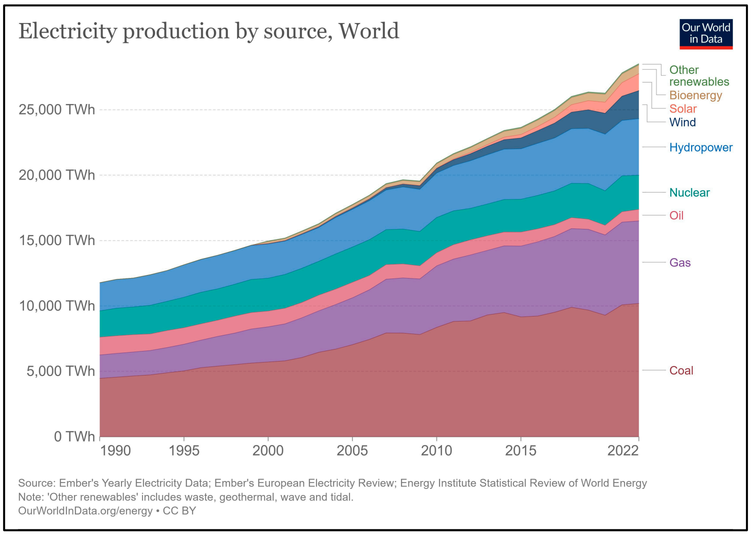

Figure S2. Electricity production (TWh) worldwide from 1990 to 2022 [183]. Source: `OurWorldInData.org`. `Note:` Terawatt hour (TWh) is a unit of energy used for expressing the amount of produced energy, electricity and heat.

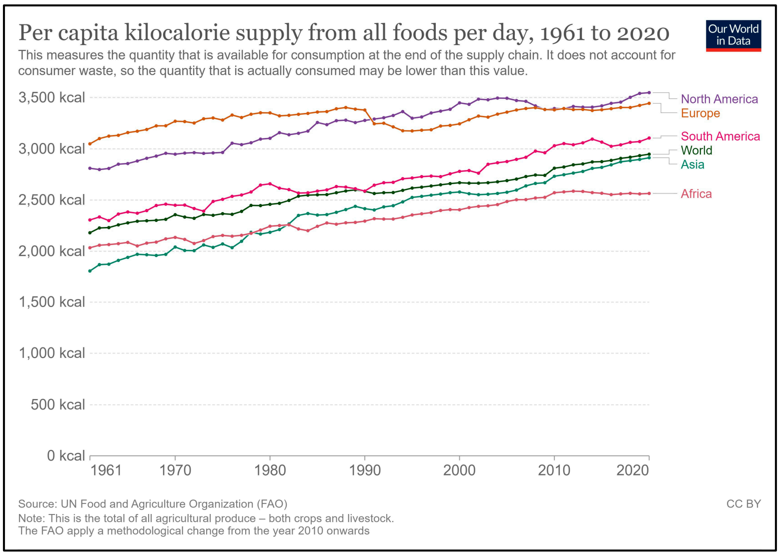

Figure S3. Per capita kilocalorie supply from all foods per day from 1961 to 2020 from different regions and worldwide [184]. Source: `OurWorldInData.org`.

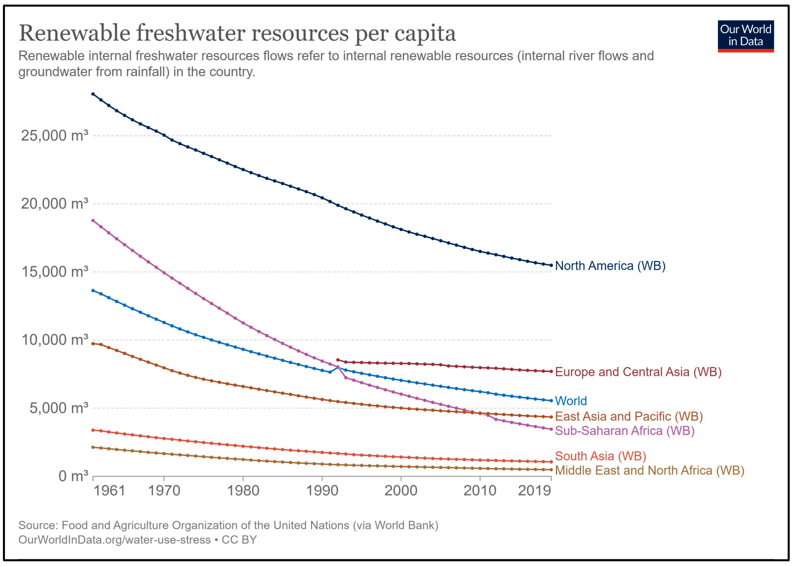

Figure S4. Renewable freshwater resources per capita of different regions in the world from 1961 to 2019 [185]. Source: OurWorldInData.org. Note: Per capita renewable resources depend on two factors: the total quantity of renewable flows and the size of the population.
